# The transcription factor ZNF469 regulates collagen production in liver fibrosis

**DOI:** 10.1172/jci.insight.182232

**Published:** 2025-02-25

**Authors:** Sebastian Steinhauser, David Estoppey, Dennis P. Buehler, Yanhua Xiong, Nicolas Pizzato, Amandine Rietsch, Fabian Wu, Nelly Leroy, Tiffany Wunderlin, Isabelle Claerr, Philipp Tropberger, Miriam Müller, Alexandra Vissieres, Lindsay M. Davison, Eric Farber-Eger, Quinn S. Wells, Quanhu Sheng, Sebastian Bergling, Sophia Wild, Pierre Moulin, Jiancong Liang, Wayne J. English, Brandon Williams, Judith Knehr, Marc Altorfer, Alejandro Reyes, Johannes Voshol, Craig Mickanin, Dominic Hoepfner, Florian Nigsch, Mathias Frederiksen, Charles R. Flynn, Barna D. Fodor, Jonathan D. Brown, Christian Kolter

**Affiliations:** 1 Novartis Biomedical Research, Basel, Switzerland.; 2 Division of Cardiovascular Medicine,; 3 Department of Surgery, and; 4 Department of Biostatistics, Vanderbilt University Medical Center (VUMC), Nashville, Tennessee, USA.; 5 Institute of Pathology, Department of Laboratory Medicine and Pathology, Lausanne University, Lausanne, Switzerland.; 6 Hospital and Lausanne University, Lausanne, Switzerland.; 7 Chief Scientific Officer, Deciphex Ltd., Dublin, Ireland.; 8 Department of Pathology, Microbiology, and Immunology, VUMC, Nashville, Tennessee, USA.; 9 Novartis Biomedical Research, Cambridge, Massachusetts, USA.; 10 Novartis Venture Fund, Basel, Switzerland.

**Keywords:** Hepatology, Inflammation, Fibrosis

## Abstract

Metabolic dysfunction–associated steatotic liver disease (MASLD) — characterized by excess accumulation of fat in the liver — now affects one-third of the world’s population. As MASLD progresses, extracellular matrix components including collagen accumulate in the liver, causing tissue fibrosis, a major determinant of disease severity and mortality. To identify transcriptional regulators of fibrosis, we computationally inferred the activity of transcription factors (TFs) relevant to fibrosis by profiling the matched transcriptomes and epigenomes of 108 human liver biopsies from a deeply characterized cohort of patients spanning the full histopathologic spectrum of MASLD. CRISPR-based genetic KO of the top 100 TFs identified ZNF469 as a regulator of collagen expression in primary human hepatic stellate cells (HSCs). Gain- and loss-of-function studies established that ZNF469 regulates collagen genes and genes involved in matrix homeostasis through direct binding to gene bodies and regulatory elements. By integrating multiomic large-scale profiling of human biopsies with extensive experimental validation, we demonstrate that ZNF469 is a transcriptional regulator of collagen in HSCs. Overall, these data nominate ZNF469 as a previously unrecognized determinant of MASLD-associated liver fibrosis.

## Abstract 2

ZNF469 is a protein that regulates how scar tissue forms in the liver in response to fat accumulation.

## Graphical abstract

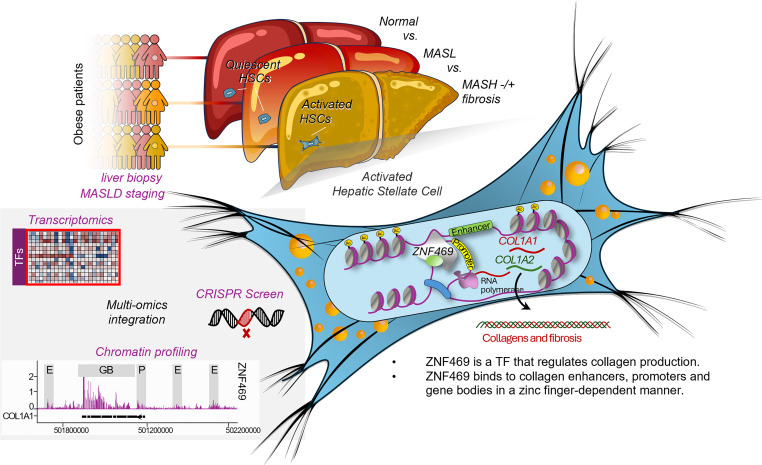

## Introduction

Metabolic dysfunction–associated steatotic liver disease (MASLD) is the most common form of liver disease in the developed world (1). The condition is closely associated with obesity and insulin resistance, often due to lifestyle factors such as high-calorie diets and sedentary behavior. MASLD exists across a spectrum of clinical and pathologic stages characterized by (a) steatosis, (b) inflammation (also referred to as steatohepatitis), and (c) fibrosis/cirrhosis (2). Morbidity and mortality from MASLD escalate during the transition to fibrosis/cirrhosis. Despite the concerning epidemiologic trends for MASLD, only one drug therapy has been recently approved for metabolic dysfunction-associated steatohepatitis (MASH) with moderate to advanced fibrosis (3, 4). Orthotopic liver transplantation is the only curative treatment for patients with late-stage MASLD. Notably, reversal of liver fibrosis has been achieved by bariatric surgery (5) and in patients with chronic Hepatitis B and C in response to long-term eradication of viruses with targeted antiviral therapies (6–8). The potential to forestall or reverse this morbid and fatal stage of disease renders liver fibrosis an intently pursued therapeutic target in MASLD.

Activated hepatic stellate cells (aHSCs) are a key cell type that drives fibrogenesis in all forms of chronic liver disease, including MASLD (9). HSCs comprise approximately 5%–10% of the nonparenchymal cell population in the liver (10). In physiologic contexts, resident HSCs exist in a quiescent state (qHSCs), serving as a storage depot for vitamin A (11). However, in response to toxins, inflammatory signals, and growth factors, HSCs become activated and assume a myofibroblast-like cell state capable of migration, proliferation and collagen production (12).

Reversible changes in cell state are orchestrated by an interplay between multiple transcription factors (TFs) and chromatin-dependent signaling pathways. In HSCs, the overall balance of activity between lineage-determining TFs (LDTFs) along with signal-responsive TFs (SRTFs) determines the inflammatory and fibrogenic gene expression programs (13–17).

More recently, occupancy maps of chromatin coactivators coupled with genome-wide enhancer profiles — e.g., measured by acetylation of histone 3 at lysine 27 (H3K27ac, a histone mark of active regulatory regions) — have been used to reconstruct transcriptional circuits involving a core set of TFs that orchestrate cell type–specific gene expression programs (18–21). Whether this conceptual framework could be used to discover TFs that drive disease-specific cell state transitions in human MASLD has never been tested. The identification and therapeutic targeting of transcriptional regulators involved in stage-specific disease progression could reveal new approaches to treat fibrotic MASH.

In this study, we integrate RNA-Seq with enhancer profiles of liver tissue obtained from patients with obesity at the time of bariatric surgery to identify candidate TFs predicted to regulate collagen expression and fibrosis. A CRISPR KO screen of these prioritized TFs in human HSCs reveals *ZNF469* — a causal gene in brittle cornea syndrome (BCS) featuring abnormal extracellular matrix production — as a regulator of subsets of collagen genes and genes involved in extracellular matrix (ECM) homeostasis. The specificity in regulation of collagen reflects direct ZNF469 binding to the genes of collagen types 1, 3, and 5. Regulation of ZNF469 is conserved in mouse stellate cells and murine models of liver fibrosis and MASLD as well as other human fibrotic diseases. Collectively, our data reveal how integration of multiomics readouts can be leveraged to gain an improved understanding of transcriptional regulators involved in the pathogenesis of HSC activation and liver fibrosis.

## Results

### Integration of transcriptomics and cis-regulatory landscapes in human MASLD livers predicts activity of TFs involved in fibrosis.

**Figure 1:**
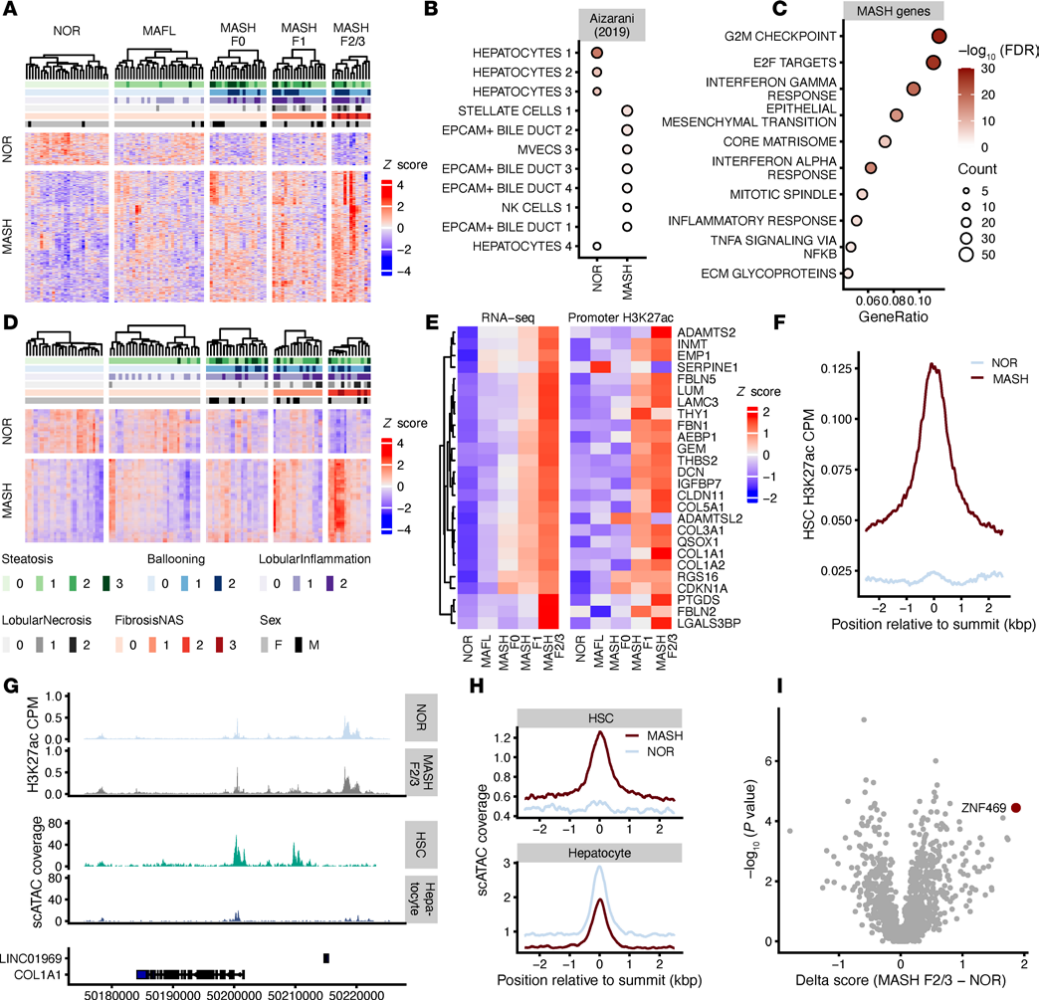
Integration of transcriptomics and *cis*-regulatory landscapes in human MASLD livers predicts activity of TFs involved in fibrosis. (**A**) Heatmap of RNA-Seq data normal versus MASH. (**B**) Enrichment plot of cell types based on RNA-Seq. (**C**) Enrichment plot of GO terms. (**D**) Heatmap of CUT&RUN H3K27ac data clustered by normal versus MASH. (**E**) Heatmap of expression across the top enriched genes in MASH versus normal concatenated by H3K27ac change at promoters. (**F**) Correlation of H3K27ac from primary HSC compared with H3K27ac from the cohort. (**G**) Genome browser view of the H3K27ac profile at the *COL1A1* locus compared with publicly available scATAC-Seq data from cultured fetal HSCs and hepatocytes. (**H**) Correlation plot comparing scATAC-Seq signal in HSCs or hepatocytes and H3K27ac signal in the MASLD cohort. (**I**) Volcano plot of predicted TF activity generated with DoRothEA analysis comparing MASH_F2/3 versus normal (NOR) samples.

To extract gene signatures in human MASLD, we characterized the transcriptomes of wedge liver biopsies collected from patients at the time of bariatric surgery. Thirty patients with MASL, 50 patients with MASH, and 28 patients without liver disease (histologically normal) were identified using MASH–Clinical Research Network (MASH-CRN) scoring criteria by a pathologist blinded to the clinical data (Supplemental Figure 1; supplemental material available online with this article; https://doi.org/10.1172/jci.insight.182232DS1). Among these 108 patients, the mean age was 43 years, BMI was 46.6 kg/m^2^, 84.3% were women, 28.7% had type 2 diabetes, 21.2% had hypercholesterolemia, 48.1% had high blood pressure, and 26.8% were current or former smokers. Regarding medication use, 33% were on a proton pump inhibitor (PPI), 28% on a selective serotonin receptor inhibitor (SSRI), 22% on hormone replacement therapy (HRT), and 26% on a nonsteroidal anti-inflammatory drug (NSAID) (Supplemental Table 1). Using this deeply characterized cohort, we identified 582 upregulated and 143 downregulated genes when comparing patients with MASH with a fibrosis score of 2 or 3 (MASH_F2/F3) to healthy/normal (NOR) liver tissue (FDR ≤ 0.01 and |log_2_FC| > 0.5; Figure 1A and Supplemental Table 2).

Having identified these signature genes between NOR and MASH_F2/F3, we next performed a gene set enrichment analysis (GSEA) on publicly available single-cell RNA-Seq (scRNA-Seq) data to ascertain whether these signatures were enriched in specific cell types in the liver (13, 14). We observed an enrichment of hepatocyte marker in genes upregulated in healthy livers, whereas HSC markers were enriched in genes upregulated in MASH livers (FDR < 0.01; Figure 1B). Activated HSCs play a known and key role in hepatic fibrosis (15). Thus, our finding that the disease-enriched signature genes mapped back to HSCs is consistent with current paradigms of liver fibrosis (17). Based on this result, we then performed pathway analysis to determine whether these genes are functionally related. The genes upregulated in MASH are enriched in pathways related to epithelial-mesenchymal transition (EMT) and core matrisome biology, both consistent with an increase in fibrogenesis (Figure 1C) and consistent with previously published work (13, 16).

### Inference of TF activity from cis-regulatory landscapes in MASLD.

MASLD is a complex disease with multiple signals integrated at the level of gene regulation and governed by the interplay between master regulatory TFs and chromatin signaling. Thus, we aimed to infer activity of TFs between MASH_F2/3 disease stage and histologically normal livers. We profiled genome-wide H3K27ac signal using CUT&RUN in 99 liver samples from the VUMC biorepository (22). Overall, our analysis identified 14,348 genome regions with differential H3K27ac abundance, when comparing the cases of MASH_F2/3 versus histologically normal livers (FDR ≤ 0.01 and |log_2_FC| > 0.5; Figure 1D and Supplemental Table 3). We found that 4,937 regions were enriched in normal livers, while 9,411 regions were associated with disease (Figure 1D). Moreover, 15 of 26 genes that were most significantly regulated on a transcriptomic and chromatin level during the transition to fibrosis (MASH_F23 versus normal) were genes linked to collagen-containing ECM (e.g., *COL1A1*, *COL1A2*, *COL3A1*, *COL5A1*, *IGFBP7*, *DCN*, *AEBP1*, *LGALS3BP*, *THBS2*, *FBLN2*, *FBLN5*, *FBN1*, *SERPINE1*, *ADAMTS2*, and *LUM*; Figure 1E). Notably, genomic regions with enriched H3K27ac signals in MASH displayed high levels of H3K27ac signal also in HSC monocultures in vitro (Figure 1F and Supplemental Figure 1E), suggesting a contribution of HSCs in the epigenetic landscape rewiring in disease development and progression. To confirm this observation, we compared our NOR and MASH_F2/3 regions to scATAC-Seq data from Zhang et al. to identify cell type–specific regulatory regions (23). We identified disease-specific peaks of H3K27ac in the promoter as well as upstream intergenic regions of the gene *COL1A1*, a core ECM component associated with fibrosis, that overlapped with scATAC peaks from HSCs (Figure 1, G and H, and Supplemental Figure 1E). We could further show that HSC-enriched ATAC-Seq signals displayed higher levels of chromatin accessibility in MASH biopsies compared with normal biopsies (Figure 1H). These results suggest that gene expression together with H3K27ac profiling capture distinct disease and normal cell states within the entire cohort.

The data demonstrate that patterns of enhancer signal and gene expression can identify disease stages, suggesting that we could use this information to infer TF activity. To test this concept, we used 6 different computational tools — ANANSE (24), CRCmapper (25), DoRothEA (26), RcisTarget (27), MonaLisa (28), and HOMER (29) — to call TFs that might explain the chromatin and transcription changes in diseased livers by pairwise comparison between normal and MASH_F2/F3 samples. In total, those tools identified 684 TFs, and more than 50% (369 TFs) were identified only with 1 method. For instance, ZNF469 showed the highest activity at MASH_F2/3 compared with normal samples according to DoRothEA (Figure 1I) but was not identified by any of the other methods (Supplemental Table 4).

### CRISPR loss-of-function screen identifies transcriptional regulators of collagen production in human HSCs.

**Figure 2:**
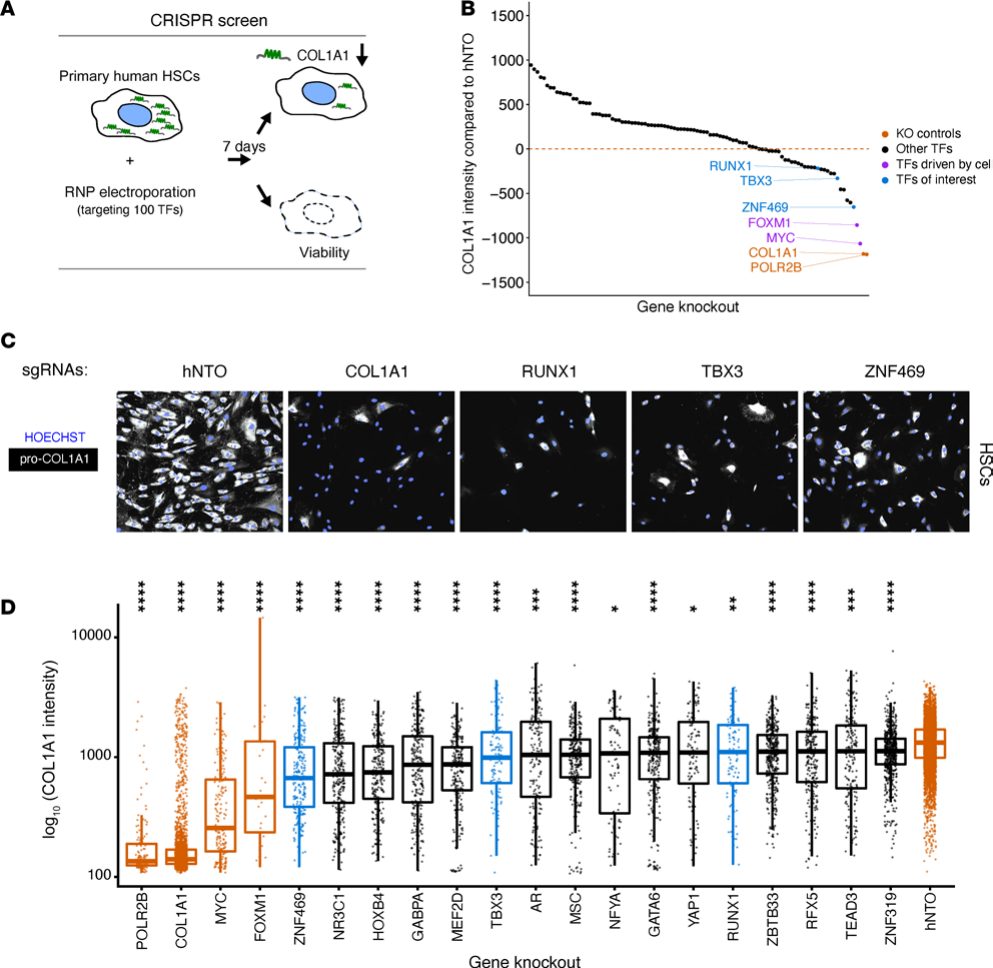
CRISPR loss-of-function screen identifies transcriptional regulators of collagen production in primary human HSCs. (**A**) Schematic of targeted CRISPR screen with single-cell high-content imaging read-out of collagen protein expression measured by immunofluorescence (IF). (**B**) Waterfall plot of primary screen results at day7. (**C**) Representative IF photomicrographs showing COL1A1 staining in the following CRISPR-KO conditions: human nontarget control (hNTO) and *COL1A1*-, *RUNX1-*, *TBX3-*, and *ZNF469-*KO. (**D**) Box plots showing distribution of COL1A1 expression upon KO of top TF hits; each dot corresponds to COL1A1 IF measurement in 1 cell. We used a Wilcoxon test to compare the medians of the COL1A1 IF expression. *P ≤ 0.05; **P ≤ 0.01, ***P ≤ 0.001, ****P ≤ 0.0001.

To validate our findings experimentally, we performed an arrayed, CRISPR loss-of-function screen focusing on 100 TFs that were selected by multiple criteria (e.g., absolute score per method, high score [i.e. hit] in multiple tools, expression in HSCs; Supplemental Tables 5–7). Given the strong links to HSCs as determined by the transcriptomics as well as enhancer profiling in patient tissues, we performed the CRISPR assays in primary human HSCs (Figure 2A). We quantified, at single-cell resolution, the difference of intracellular COL1A1 between the CRISPR-derived RNA (crRNA)/ trans-activating CRISPR RNA (tracrRNA) complexes (for simplicity, these complexes will be referred to as sgRNA) targeting each TF compared with the human nontargeting oligonucleotide (hNTO) sgRNA controls. We identified 18 TFs that downregulated COL1A1 (Figure 2, B–D) compared with the median COL1A1 expression of the hNTO (threshold < –200; Figure 2B). Since cell death could also decrease intracellular COL1A1, the number of cells was used to determine viability. We observed that our cell viability controls (such as POLR2B) and predicted or previously identified essential TFs in myofibroblasts, such as MYC and FOXM1 (30), decreased cell viability and were discarded from further experiments (Supplemental Figure 2). Therefore, we nominated 11 candidate TFs for further validation, including ZNF469 as the top hit.

### ZNF469 KO alters collagen mRNA expression in HSCs.

**Figure 3:**
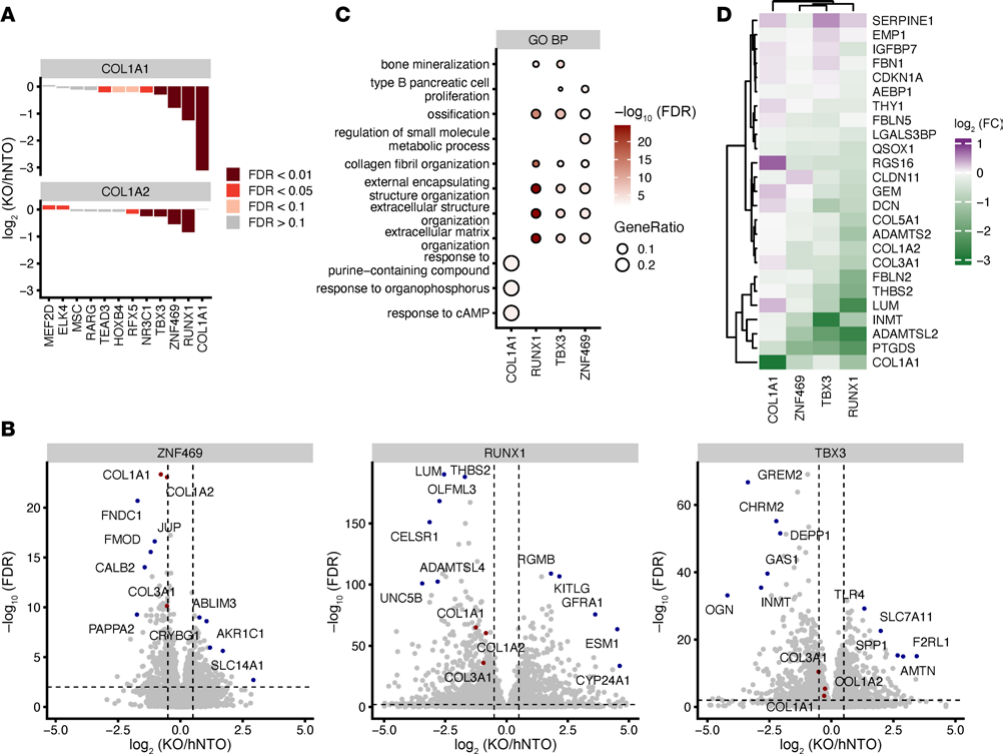
ZNF469-KO alters collagen mRNA expression in HSCs. (**A**) Bar plot of *COL1A1* and *COL1A2* gene expression obtained by RNA-Seq, after KO of top hits from the CRISPR screen: *MEF2D*, *MSC*, *RARG*, *RFX5*, *HOXB4*, *TEAD3*, *NR3C1*, *TBX3*, *ZNF469*, *RUNX1*, *ELK4*. (**B**) RNA-Seq volcano plots for *ZNF469*, *TBX3*, and *RUNX1* with *COL1A1* and *COL1A2* highlighted. (**C**) Gene set enrichment of downregulated genes upon *ZNF469*, *RUNX1*, or *TBX3* KO. (**D**) Heatmap of differential HSC genes (between MASH_F2/3 and normal) from Figure 1E upon CRISPR KO with *z* score distribution across hNTO, *COL1A1*, *ZNF469*, *TBX3*, and *RUNX1*.

To further prioritize the top candidates, we performed RNA-Seq experiments in order to: (a) filter out factors that regulate COL1A1 protein levels posttranscriptionally, (b) measure the specificity of the transcriptional effects on the whole gene signature compared with global effects, and (c) control CRISPR editing efficiency of each target (Supplemental Figure, 3 A and B). The latter point is particularly important for genes such as *ZNF469,* since its transcript harbors one large coding exon and CRISPR mutations do not lead to nonsense-mediated mRNA decay as previously demonstrated for the mouse homolog (31). Notably, only the targeting of 3 TFs (*ZNF469*, *RUNX1*, *TBX3*) resulted in a significant (FDR < 0.01) downregulation of *COL1A1* mRNA (Figure 3A). All these TF perturbations also downregulated *COL1A2,* located on a different chromosome from *COL1A1* (Figure 3A), suggesting that they are upstream regulators of type 1 collagens. Compared with *RUNX1* KO and *ZNF469* KO, *TBX3* KO had the smallest effect on *COL1A1* and *COL1A2* mRNA (Figure 3B). When considered globally, similar gene sets were enriched for *ZNF469*, *RUNX1*, and *TBX3* KO including ECM organization (Figure 3C and Supplemental Figure 3C). In particular, for *RUNX1* KO, the 2 top significantly downregulated genes were *LUM* and *THBS2*. For *ZNF469* KO, the 2 most significantly downregulated genes were *COL1A1* and *COL1A2*, and we confirmed this in a second donor (Figure 3B and Supplemental Figure 3E). All 4 genes (*LUM*, *THBS2*, *COL1A1*, and *COL1A2*) are part of the collagen-containing ECM GO term (GO: 0062023). Since the disease-specific upregulated transcripts in liver samples also contained many ECM genes, we explored whether these TFs reduce expression of genes in the signature, restricting our analysis to targets that were expressed in HSCs (Figure 1E). While both *TBX3* and *RUNX1* showed a strong downregulation of most of these genes, *ZNF469* KO had a more specific effect on *COL1A1*, *COL1A2*, and *COL3A1* as well as *INMT*, *ADAMTSL2*, and *PTGDS* (Figure 3D and Supplemental Figure 3D). In addition to this HSC-specific panel, *ZNF469* KO also significantly downregulated *FMOD* and *FNDC1*, 2 other fibrosis-related genes (Figure 3B) (32).

### ZNF469 localizes to gene bodies of collagen genes in HSCs.

**Figure 4:**
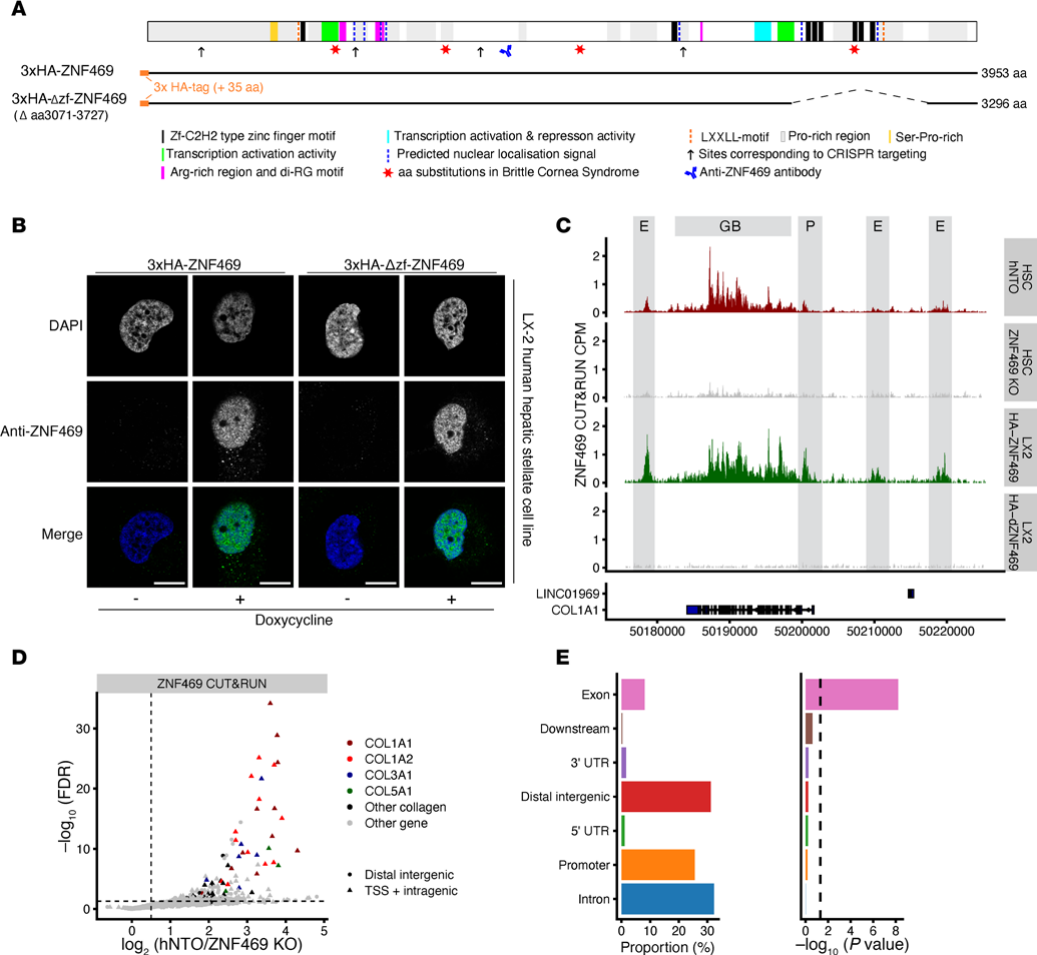
ZNF469 is enriched at extracellular matrix–related genomic sites. (**A**) Schematic of ZNF469 protein (NP_001354553.1) with previously reported and predicted sequence features, regions active in transactivation domain reporter assays, amino acid substitutions linked to the BCS, anti-ZNF469 antibody antigen region, and positions corresponding to the CRISPR sgRNA target sites. Coverage of ZNF469 by full-length and deletion-harboring cDNA constructs used for stable inducible cell line generation are shown (see also Supplemental Table 8). (**B**) Representative photomicrographs of ZNF469 subcellular localization by indirect immunofluorescence staining with the anti-ZNF469 antibody in overexpressing transgenic LX-2 cells. Scale bar: 10 mM. (**C**) Genome browser tracks at the *COL1A1* locus of ZNF469 CUT&RUN signals generated with the anti-ZNF469 antibody in CRISPR experiments in nontargeting and *ZNF469-*targeted human HSCs as well as transgenic LX-2 cells with doxycycline-inducible full-length or deletion-harboring ZNF469 cDNA (E, putative enhancer; P, promoter; GB, gene body). (**D**) Volcano plot of differential occupancy of endogenous ZNF469 in control cells versus ZNF469-KO cells. Data shows change in signal at distal elements (circles) and TSS/intragenic sites (triangles) with color coded for collagen genes. (**E**) Bar plots of distribution of ZNF469 peaks at genomic annotations.

The CRISPR screen and mRNA profiling as well as recently published results (33, 34) suggest that ZNF469 plays a selective role in the regulation of collagen gene expression. However, there is a lack of functional data demonstrating that it is a bona fide DNA binding protein. Thus, we chose to explore its biological role in HSCs more fully. We annotated its sequence with previously reported and protein-sequence analysis–based features that were in agreement with putative TF-, nucleic acid–, and protein-binding functions (Figure 4A) (35–38). To address questions about ZNF469 function, we designed a doxycycline-inducible piggyBac transposon system to express the full-length gene with an N-terminal 3xHA epitope tag (HA-ZNF469) and another construct (HA-Δzf-ZNF469) with a deletion of 6 of the 8 zinc fingers (ZF) (del:aa3071-3727; known hereafter as “ZF domain”) (Figure 4A and Supplemental Table 8). For practical reasons, we turned to the LX-2 cell line, which is an immortalized human stellate cell line that recapitulates features of the activated HSC. IHC results with an anti-ZNF469 antibody indicated that ZNF469 localized exclusively to the nucleus in LX-2 cells after doxycycline treatment (Figure 4B). Motivated by this result, we next mapped the genome-wide binding sites of endogenous ZNF469 in HSCs using CUT&RUN with the same antibody and in the transgenic LX-2 cells. Cistromic analyses demonstrated a specific gene-body distribution of ZNF469 at collagen genes, including *COL1A1* (Figure 4C), *COL1A2*, and *COL3A1* (Supplemental Figure 4A). In particular, ZNF469 was bound to the promoter of *COL1A1* and several putative enhancer elements in proximity to this gene. No DNA binding of ZNF469 was observed at these loci upon KO of endogenous ZNF469 in HSC. Moreover, LX-2 cells expressing the deletion construct also did not show any DNA binding providing important controls for the antibody and CUT&RUN overall (Figure 4C and Supplemental Figure 4A). By differential analysis of chromatin profiles in control cells versus ZNF469-KO cells, the ZNF469 signal was significantly higher at intragenic sites including transcriptional start sites of collagen genes (Figure 4, D and E).

We next tested whether ZNF469 overexpression could rescue the *COL1A1* reduction when knocking out the endogenous *ZNF469* gene. For these series of experiments, we first generated mouse stellate cell lines (JS1) targeting the mouse ortholog *Zfp469* using CRISPR guides against either the proximal portion of the gene or a region encoding for the ZF domain near the 3′-end of the gene. Both CRISPR treatments resulted in a ~50% reduction in *Col1a1* and *Col1a2* mRNA in these cells as compared with nontargeting control (Supplemental Figure 4B). Introduction of full-length HA-tagged human ZNF469 rescued the *Col1a1* and *Col1a2* mRNA levels and even increased *Col1a1* mRNA levels 2- to 4-fold compared with baseline levels. In addition, an orthogonal approach using transient siRNA knockdown of *Zfp469* resulted in a ~50% reduction in *Col1a1* and *Col1a2* mRNA when compared with a scrambled siRNA control (Supplemental Figure 4C). These results suggest conservation of *ZNF469/Zfp469* as a regulator of collagen expression between human and mouse species.

### ZNF469 KO alters local chromatin structure at collagen loci in HSCs.

**Figure 5:**
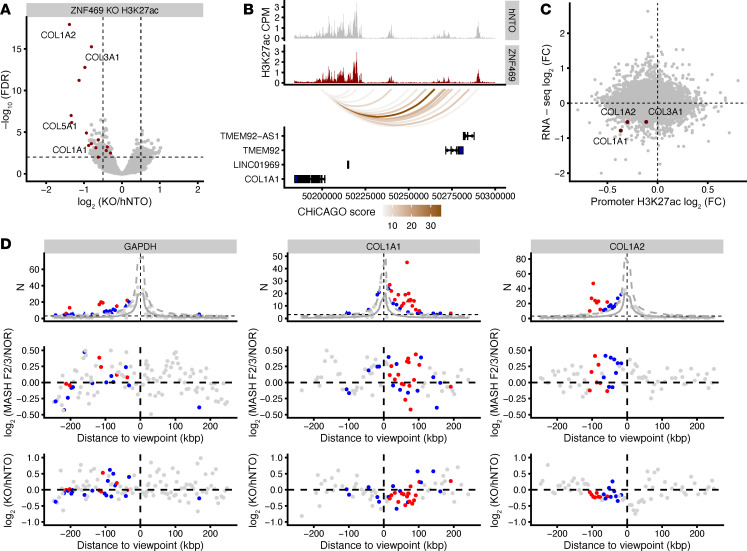
ZNF469 KO alters local chromatin structure at collagen and ECM loci in HSCs. (**A**) Volcano plot of CUT&RUN H3K27ac with *COL1A1* and *COL1A2* highlighted upon *ZNF469* KO. (**B**) Genome track of H3K27ac occupancy and Promoter Capture Micro-C at the *COL1A1* locus. (**C**) RNA-Seq and CUT&RUN integration upon *ZNF469* KO highlighting *COL1A1* and *COL1A2* as the top-affected genes. (**D**) Three-dimensional chromatin interactions. Top panel: Promoter Capture Micro-C data showing promoter bait plots for *GAPDH*, *COL1A1*, and *COL1A2*. Interactions with a Chicago score ≥ 5 were considered significant (red dots); subthreshold interactions (3 ≤ score < 5) are shown as blue dots (64). Gray lines show expected counts, and dashed lines the upper bound of the 95% CI. Middle panel: H3K27ac signal of disease versus normal; colors refer to top panel. Lower panel: H3K27ac human primary HSC cell signal upon *ZNF469* KO; colors refer to top panel.

To further confirm ZNF469 as a regulator of collagen expression, we profiled genome-wide H3K27ac following *ZNF469* CRISPR KO (Figure 5A). *ZNF469* KO resulted in 171 differential H3K27ac peaks (FDR ≤ 0.01 and |log_2_FC| > 0.5). As shown with ZNF469 binding*, COL1A1* and *COL1A2* had been among the loci with the greatest reductions in H3K27ac (Figure 5, A and B, and Supplemental Figure 5A). In particular, the *COL1A2* locus was the most significantly differentially acetylated gene with multiple signals spanning the gene-body (Supplemental Figure 5A). These results suggest that ZNF469 has a specific role in regulating collagen type I genes. Notably, other collagens (e.g., *COL3A1*, *COL5A1)* were also among the top 10 affected loci (Figure 5A). By integrating the RNA-Seq and H3K27ac datasets, we observed that the downregulation of *COL1A1* and *COL1A2* mRNA correlates with a reduction of H3K27ac at those sites using promoter or enhancer annotation (Figure 5C and Supplemental Figure 5, B and C). Of note, this pattern is the inverse of what was observed with the human liver samples in which ZNF469 was predicted to be more active and supports a directionally consistent link between ZNF469 action/expression and collagen expression (Figure 1E). In addition, ZNF469 was not bound to chromatin at the genomic loci of canonical TGF-β–inducible HSC marker genes, except for collagen targets (Supplemental Figure 5C). This suggests that the expression of other HSC activation markers was not directly affected by *ZNF469* KO. Moreover, TGF-β did not consistently induce *ZNF469* mRNA expression in HSCs or tissue fibroblasts (Supplemental Figure 6, A–D) (39–42). Since enhancer-dependent signaling can occur over long distances through chromatin looping to promoters, we next explored the 3-dimensional gene regulatory landscape in HSCs using Promoter Capture Micro-C. Promoter Capture Micro-C is a chromatin conformation assay that fragments DNA with Micro-Coccal nuclease and then uses capture probes to pull down human promoters from the sequencing library. This approach allowed us to discover interactions between distal regulatory elements and human promoters in an unbiased way across large regions of the genome. Using WT primary HSCs, we observed significant interactions of distal regions, likely enhancers, to the promoters of *COL1A1* and *COL1A2*, respectively (Figure 5, B–D, and Supplemental Figure 5, A and B). Notably, a subset of these interactions overlapped with H3K27ac signals in human livers with MASLD (Figure 5D) and are altered in HSCs in vitro upon *ZNF469* KO (Figure 5D).

In summary, activity of enhancers at the *COL1A1* and *COL1A2* loci, as indicated by H3K27ac signal, were reduced following *ZNF469* KO, and chromatin conformation assays revealed likely interactions between these same regions and promoters of the collagen genes that are downregulated in response to *ZNF469* KO. Overall, these data provide further evidence that ZNF469 functions as a transcriptional regulator of collagen gene expression through enhancer-dependent signaling in HSCs.

### ZNF469 expression correlates with fibrosis in human MASLD, pulmonary disease, and murine models of fibrotic liver disease.

**Figure 6:**
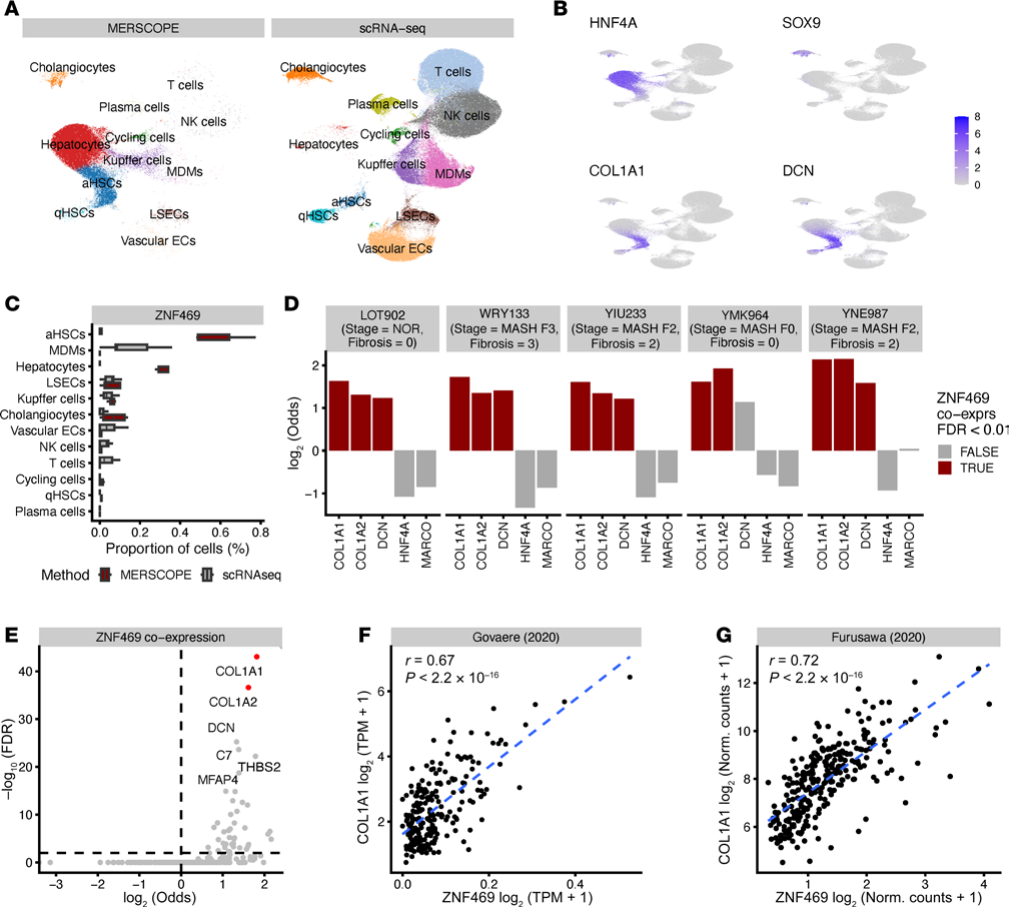
ZNF469 expression correlates with collagen production in HSCs in human MASLD. (**A**) Integrated MERSCOPE and scRNA-Seq UMAP showing liver cell types. (**B**) Integrated UMAP embedding colored by marker gene expression for following cell types: Hepatocytes *HNF4A*, Cholangiocytes *SOX9*, activated hepatic stellate cells *COL1A1* and *DCN*. (**C**) *ZNF469* expression per identified cell type in MERSCOPE and scRNA-Seq. (**D**) *ZNF469* is coexpressed with *COL1A1*, *COL1A2*, and *DCN* but not *HNF4α* or *MARCO*. (**E**) *COL1A1* and *COL1A2* are the top 2 coexpressed genes with *ZNF469* across all patients. (**F**) Scatter plot showing correlation (Pearson) of *ZNF469* and *COL1A1* expression in public MASH cohort. (**G**) Scatter plot showing correlation (Pearson) of *ZNF469* and *COL1A1* expression in published interstitial pulmonary fibrosis cohort.

ZNF469 loss-of-function decreased both *COL1A1* and *COL1A2* expression, which encode 2 collagen proteins known to form heterotrimers. Thus, regulation of both genes by ZNF469 might modulate levels of fibrosis at the organ level. To explore this relationship, first we performed multiplexed RNA-FISH in a subset of liver sections from the VUMC liver biorepository using MERSCOPE technology (Figure 6A). For this assay, we designed a 300-gene probe set that included markers of aHSCs (e.g., *DCN*) and other cell types including hepatocytes (e.g., *HNF4a*) along with *COL1A1* and *ZNF469*. MERSCOPE enabled simultaneous measurements of the number and spatial distribution of *ZNF469* transcripts along with other cell marker genes in normal versus fibrotic MASH human livers (Figure 6, A–C; Supplemental Figure 7, A–C; Supplemental Figure 8; and Supplemental Table 9). We detected coexpression of *ZNF469* with *COL1A1, COL1A2,* and *DCN* (Figure 6, D and E, and Supplemental Figure 8). Notably, we did not observe coexpression of *ZNF469* with *HNF4A* (hepatocyte marker) or *MARCO* (Kupffer cell marker) (Figure 6D). Hence, we demonstrated *ZNF469* transcript expression was localized in HSCs in human MASLD livers with fibrosis from the VUMC repository.

The specificity in regulating collagen type I is also in agreement with its expression being restricted to a subset of cell types across the human body. The highest expression of *ZNF469* was observed in different fibroblasts (Supplemental Figure 8). We therefore wanted to explore whether the relationship between *ZNF469* expression and degree of fibrosis can be generalized using multiple additional clinical cohorts. Indeed, we observed in 6 of 6 publicly available MASLD as well as interstitial pulmonary fibrosis (IPF) cohorts a strong correlation of *COL1A1* and *ZNF469* expression (MASLD: Govaere *R* = 0.67, Hoang *R* = 0.79, Suppli *R* = 0.4; IPF: Furusawa *R* = 0.72, Konigsberg *R* = 0.56, Sivakumar *R* = 0.83; *P* < 0.01; refs. 43–48) (Figure 6, F and G, and Supplemental Figure 9, C and D).

Conservation of regulation is one way to determine potential biological relevance of predicted gene regulatory networks. We observed that *ZNF469/Zfp469* was significantly induced in 2 murine models of steatosis with fibrosis: Gubra Amylin-NASH (GAN) diet and choline-deficient, amino acid–defined high–fat diet (CDAHFD; Supplemental Figure 10A). The increase in *Zfp469* was accompanied by an increase in *Col1a1* mRNA. We did not observe a difference in *Zfp469* or *Col1a1* expression in ethionine-treated *ob/ob* mice, a distinct model of MASLD that features microvesicular steatosis and inflammation (Supplemental Figure 10A). Using RNA-FISH (RNAScope) in GAN animals, we found that *Zfp469* mRNA colocalized with *Col1a1* and *Acta2* transcripts, indicative of aHSCs (Supplemental Figure 10B) and in agreement with the human MERSCOPE data (Figure 6, D and E). We broadened this analysis to include other public cohorts (49, 50) of mouse data and confirmed that *Zfp469* mRNA correlated with *Col1a1* expression, and its expression increased upon disease progression in liver disease induced by carbon tetrachloride^–^ (CCl_4_^–^) as well as bleomycin-induced lung disease (Supplemental Figure 10, C and D). Taken together, ZNF469 is a disease-relevant, evolutionarily conserved and generalizable regulator of collagens.

## Discussion

The transition to fibrosis represents a clinically important inflection point in MASLD morbidity and mortality in humans. We discovered transcriptional regulators of fibrosis in aHSCs using multiomics on human liver samples and a CRISPR genetic screen in a primary cell model. From 100 profiled TFs, we found ZF 469 (*ZNF469*) as the top hit in regulating collagen type I protein. Follow-up mechanistic studies at RNA, chromatin, and protein levels demonstrated that ZNF469 functions as a positive transcriptional regulator of collagens and matrix genes — a finding conserved in mouse HSCs and in 2 murine models of MASLD/fibrosis. In human MASLD, liver expression of *ZNF469* was positively correlated with the extent of fibrosis. Collectively, these data demonstrate that ZNF469 is a previously unrecognized transcriptional regulator of fibrogenesis in HSCs and MASLD.

As a member of the C2H2 ZF protein family, ZNF469 is predicted to function as a TF (51). Furthermore, the protein structure possesses multiple nuclear localization sequences and putative transcriptional activation/repression domains. In our study, 5 lines of experimental evidence strongly indicate that ZNF469 functions as a TF: (a) it is expressed exclusively in the nucleus; (b) CRISPR deletion of *ZNF469* reduces collagen gene expression; (c) the protein localizes to chromatin at collagen gene loci in a ZF-dependent manner; (d) *ZNF469* KO reduces H3K27ac signals in gene bodies and at distal regulatory elements; (e) overexpression of ZNF469 rescues collagen mRNA expression in ZNF469-depleted mouse and human cell lines.

Notably, point mutations in *ZNF469* cause BCS and Ehlers-Danlos syndrome (EDS) in humans (52, 53). Both are developmental disorders that feature organ dysfunction from loss of collagen in the eye and musculoskeletal system, respectively. Moreover, deletion of *znf469* in developing zebrafish decreases synthesis of collagen and proteoglycans (54), and KO of *Zfp469* in mice recapitulates BCS with decreased collagen deposition in the cornea (31). No liver phenotypes in the KO mice were described, and no adverse effects on viability or fertility were observed (31). These data indicate substantial evolutionary conservation of ZNF469 function in the transcriptional regulation of collagen during development.

Two other TFs prioritized by the CRISPR screen and the multiomics validation data were *RUNX1* and *TBX3*. A recent study revealed that RUNX1 plays an important role in HSC activation and promotes liver fibrosis in a CCl_4_ mouse model by activating TGF-β/SMAD2/SMAD3 signaling directly (55). Our study confirms that RUNX1 has a profibrotic role in primary human HSCs. Similarly, we also found that TBX3 may be a regulatory factor in HSCs, a finding that could expand its role beyond hepatocytes in which *Tbx3* KO was recently found to protect against high-fat diet–induced MASLD in mice (56). In our study, we inferred TF activity using multiple computational tools with paired RNA-Seq and enhancer profiles as input datasets. Other groups have leveraged global and single-cell gene expression profiles and circulating proteomics in human MASLD to gain insight into pathways involved in fibrosis (43, 57). Across these studies, transcriptomic data consistently stratifies samples by disease stage. Pathways of inflammation, lipid metabolism, and ECM are all highly represented. Notably, pair-wise analysis across disease stage groups in 1 study also enabled identification of a “gene signature” that includes *COL1A1* and *COL1A2* (43) — both regulated by ZNF469. However, ZNF469 was not specifically described in these studies. All these datasets support the concept that defined molecular mediators likely control key transitions during liver disease progression. Our study adds to this growing body of work by illustrating the computational power of using multiple tools analyzing paired RNA-Seq and chromatin datasets to identify regulatory TFs in human MASLD fibrosis.

In addition to promoter and enhancer elements, ZNF469 bound selectively to collagen gene bodies, where H3K27ac signal was also most significantly lost upon *ZNF469* KO. To our knowledge, only PRDM5, in which mutations also cause BCS, has been shown to have such a specific genome occupancy profile at collagens genes (58). The mechanisms to explain how ZNF469 binds collagen genes remains unclear. No consensus DNA motif has yet been described for this TF in the literature, and the large regions of DNA occupancy at gene bodies in our datasets made it more challenging to identify discrete DNA binding sequences. Whether pathogenic variants in ZNF469 causing BCS affect DNA binding directly will be interesting to test as a potential transcriptional mechanism for the collagen downregulation observed in the disease. TFs (e.g., YY1, KLF, MYC, and GATA2) can bind to RNA through arginine-rich domains (59). Thus, structural features of target genes at the RNA level could be determinants of ZNF469 localization. Previous work has also shown that PRDM5 binds to chromatin at collagen gene bodies through direct interactions with RNA polymerase II (Pol II) (58, 60). PRDM5 and ZNF469 may functionally interact at these gene loci to control Pol II–dependent transcription of collagen.

Our study has limitations. Biopsy samples were taken from individuals undergoing gastric bypass surgery. Consequently, all patients have some degree of systemic metabolic dysfunction associated with obesity including those with normal histology. Furthermore, the demographics, in particular sex and race, are not equally distributed across groups, thereby missing genetic variability across different ethnic groups. A limitation of our CRISPR screen was the focus on TFs. Another group recently performed a genome-wide CRISPR screen to identify TGF-β–responsive mediators of HSC activation, but did not report any results related to *ZNF469* KO (61). However, in our screen, an important filtering step was inclusion of TFs identified through analysis of RNA-Seq and enhancer landscapes derived directly from human liver samples. Thus, we believe the hits in our screen do have immediate translational relevance to human disease.

Conceptually, TFs are appealing therapeutic targets because of the potential to alter expression and activity of multiple genes in a disease pathway simultaneously in MASLD. However, TFs are not readily accessible for drug development due to lack of small-molecule binding pockets and presence of intrinsically disordered functional domains (62). Nuclear receptors (NRs) (e.g., PPARs, FXRs, LXRs, THRs) are a notable exception. Results of drug studies targeting NRs have been mixed. However, a clinical trial recently demonstrated that a thyroid receptor β agonist can reduce fibrosis score in MASLD (4). Our results indicate that targeted loss-of-function/inhibition of ZNF469 in HSCs should improve liver fibrosis, and future studies using preclinical models will directly test this hypothesis. Overall, our work does illuminate the power of a reverse translation approach to discover a previously unrecognized transcriptional mediator governing stage-specific progression of human MASLD.

## Methods

Supplemental Methods are available online with this article.

### Sex as a biological variable.

Both male and female individuals were included in this study. The study did include more women overall (84%) due to the nature of the referral population for bariatric surgery at VUMC (additional information in Supplemental Table 1). In the sequencing analysis, sex was included as a covariate.

### Demographic information.

The self-reported race of individuals from which liver biopsies were used in this study were as follows: 91 White, 15 African American, and 2 Hispanic individuals (Supplemental Table 1).

### Patients and sample collection.

Wedge liver biopsies of the left lateral lobe of the liver were obtained at the time of elective bariatric surgery in both male and female patients. Liver histology was reported by a minimum of 2 pathologists with experience in reporting hepatic histopathology. Hepatic steatosis, ballooning, lobular inflammation, and fibrosis were reported in accordance with the MASH-CRN scoring criteria (63). Definite histological MASH was defined as NAFLD activity score (NAS) ≥ 5 while borderline MASH was defined as NAS of 3–4. Significant and advanced fibrosis were defined as ≥ F2 and ≥ F3, respectively. Inclusion criteria were: a scheduled bariatric surgery, obesity ≥ 40 kg/m^2^ or ≥ 35 kg/m^2^ and 1 comorbidity (type 2 diabetes [fasting blood glucose ≥ 120 mg/dL; HbA1C ≥ 6.5%]; known fatty liver disease, hypertension, cardiovascular disease, or hyperlipidemia. Patients were excluded if they had prior bariatric surgery, malignancy (< 5 years), had known history of intestinal disease, malabsorptive syndrome, were pregnant or breastfeeding, established organ dysfunction, had renal disease, had α1 antitrypsin disease, had Wilson’s disease, had viral hepatis, had alcoholic liver disease, or smoked > 7 cigarettes per day.

### Animal models.

For mouse studies, C57BL/6J (Jackson Laboratory, strain no. 000664) and *ob/ob* (B6.Cg-Lep^ob^/J, strain no. 000632) were used. Both males and females were used for C57BL/6J experiments. For *ob/ob* experiments, only male mice were studied because ethionine is more toxic in females. Animals were 8–10 weeks of age at the start of GAN diet or ethionine treatment. Diets used in mouse studies: (a) GAN diet (40% fat [palm oil], 40% carbohydrates [20% fructose], 2% cholesterol; Research Diets, D09100310); (b) control diet for GAN (Research Diets, D09100304); (c) methionine-choline deficient (MCD) diet, with 60% fat (Research Diets, A06071302); and (d) control diet for MCD (Research Diets, A06061314). Ethionine (E5139) was purchased from MilliporeSigma and included in the drinking water of *ob/ob* mice at 0.15% for 1 week.

### Sources of cell lines.

Primary human stellate cells were obtained from Lonza (donor 1: HUCLS, lot HSC190131; donor 2: BioIVT, batch no. S00354, lot OTL; donor 3: Lonza HUCLS, lot 1HSC180141). LX-2 and JS1 cells were gifts from Youngmin Lee (VUMC).

### Statistics.

Primary data, sequences, and key reagents are presented in Supplemental Tables 1–11. For the CRISPR data, we used a Wilcoxon test to compare the medians of the COL1A1 immunofluorescence expression. Quantitative PCR data (JS1 CRISPR, JS1 siRNA, HSC CRISPR) were analyzed with 1-way ANOVA and Dunnett’s multiple-comparison test represented using GraphPad Prism 10.0 software. *P* ≤ 0.05 was considered significant. Sequence data analysis was performed using R 4.2.1. Pearson correlation between ZNF469 and COL1A1 was computed using cor.test function in R, with log2 transformed TPM from bulk RNA-seq as input. Additional details of statistical tests used for each experiment are described in Methods.

### Study approval.

Participants gave informed written consent before participating in this study, which was approved by the IRB of Vanderbilt University (nos. 090657 and 171845) and registered at ClinicalTrials.gov (NCT00983463 and NCT03407833). All studies were conducted in accordance with NIH and institutional guidelines for human research. The study protocol conformed to the ethical guidelines of the 1975 Declaration of Helsinki, as reflected in a priori approval by VUMC.

### Data availability.

Public studies used for this analysis are listed in Supplemental Table 12. RNA-Seq and chromatin data are available as indicated in Table 1.

## Author contributions

Conceptualization was contributed by MF, CRF, BDF, JDB, and CK. Methodology was contributed by SS, FW, NL, NP, DE, TW, A Rietsch, IC, PT, JK, MA, MM, LMD, QS, DPB, YX, PM, AV, JV, A Reyes, SB, SW, JL, WJE, BW, JDB, CRF, BDF, EFE, QSW, and CK. Investigation was contributed by SS, JDB, CRF, BDF, and CK. Visualization was contributed by SS, BDF, and CK. Funding acquisition was contributed by MF, JDB, and CRF. Supervision was contributed by MF, CM, DH, and FN. Writing of the original draft was contributed by JDB and CK. Review and editing of the manuscript was contributed by SS, JDB, CRF, BDF, and CK.

## Supplementary Material

Supplemental data

Supplemental tables 1-12

Supporting data values

## Figures and Tables

**Table 1:**
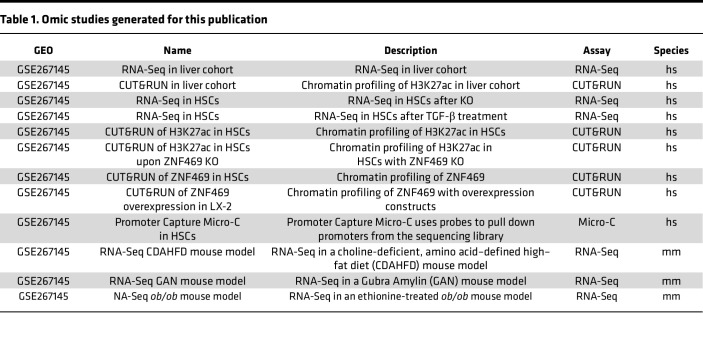
Omic studies generated for this publication

## Version 1 02/25/2025

In-Press Preview

## Version 2 04/08/2025

Electronic publication
