# Supplementary material for: The transcription factor ZNF469 regulates collagen production in liver fibrosis
Source: JCI Insight. 2025 Feb 25;10(7):e182232. doi: 10.1172/jci.insight.182232 (PMC11981625; doi:10.1172/jci.insight.182232)
Supplement: Supplemental data [file jciinsight-10-182232-s127.pdf]

List of Supplemental Materials

Supplemental Methods

Supplemental Figures 1-10

Supplemental Tables 1-12:

Supplemental Table 1: Clinical characteristics of the study population

Supplemental Table 2: Differential genes for all RNA-seq datasets

Supplemental Table 3: Differential peaks for all CUT&RUN datasets

Supplemental Table 4: TF tool results

Supplemental Table 5: CRISPR guides

Supplemental Table 6: CRISPR raw results

Supplemental Table 7: CRISPR statistical results

Supplemental Table 8: ZNF469 sequence information

Supplemental Table 9: MERSCOPE sample information and probes

Supplemental Table 10: Proteomics

Supplemental Table 11: Key reagents

Supplemental Table 12: Public omic studies

Supplementary Methods

Study Design

This study was designed to map and elucidate altered transcriptional circuitries and transcription factor pathways that define the disease states and transitions from healthy to MASLD and ultimately fibrosis. The outcome of this study are transcription factors that are relevant in long term disease progression accompanied by a deepened understanding of transcriptional landscape and regulation in chronic progressive liver disease at clinically relevant time points.

Cell culture

All cells were cultured at 37°C in 5% CO_2 in humidified incubators and were free from mycoplasma (MycoAlert Detection Kit, Lonza). Human primary hepatic stellate cells used for the main experiments were from Lonza (donor 1: HUCLS, lot HSC190131, female, Caucasian, age = 38 years; BMI = 31.2 kg/m^2). Two additional donors were used for confirmation experiments (donor 2: BioIVT, batch no: S00354, Lot: OTL, male, Caucasian, age = 49, BMI = 26.7 kg/m^2; donor 3: Lonza HUCLS, lot 1HSC180141, female, African American, age = 11 years, BMI = 35.2 kg/m^2). Cells were thawed according to the supplier's instructions and expanded in collagen-coated flasks in human stellate cell growth medium (Lonza, MCST250) supplemented with 50 mM 2-Phospho-L-ascorbic acid (Merck, 49752). Frozen stocks were prepared at passage 4 and used for all subsequent experiments. Typically, cells were thawed and passaged once before electroporation at passage 6. For CRISPR validation experiments cells were seeded in triplicates directly after electroporation, were split at day 7 or 8 into a bigger format, medium was changed at day 4 and day 11 and cells were collected at day 12. For RNA-seq experiments, cells were seeded in triplicates in collagen-coated 24 well plates (5000 cells / well) and split into the well of a collagen-coated 6 well plate at day 7. For profiling experiments, cells were first seeded in triplicates in collagen-coated 10 cm dishes after electroporation. To compensate for the differences in cell growth effect of each target, the number of cells plated / 10 cm dish were 166,000 for *RUNX1* and *TBX3*; 100,000 for hNTO, *ZNF469* and *COL1A1*. Each of the 10 cm dish replicates was counted and re-seeded into a collagen-coated T150 flask at day 8 (800,000 cells / flask for *RUNX1* and *TBX3*, 600,000 cells / flask for hNTO, *ZNF469* and *COL1A1*. For TGF-β treatment experiments, primary HSCs were thawed

in MCST250 medium without 2-Phospho-L-ascorbic acid and then seeded in 6 well plates in DMEM medium (Gibco, 1966-021) containing 0.4% FBS (Gibco, 26140-079). The medium was changed the following day and cells were incubated in the same low FBS medium with addition of 5 and 10 ng/ml TGF-β (Gibco, PHG9214), for 24h (qPCR), 4 and 7 days (RNA-seq, only 5 ng/ml). LX-2 cells (SCC064, Merck) were cultured with 10% FBS medium without 2-Phospho-L-ascorbic acid before switching to 0.1% serum ON followed by the addition of 10 ng/ml TGF-β for 6 or 24 hours in the same low serum medium. For stable inducible cDNA expression experiments LX-2 cells were cultured in DMEM supplemented with 1% penicillin/streptomycin (Gibco 15070063), 50 mM 2-Phospho-L-ascorbic acid and 2% TET-system-approved FCS (Gibco A4736201). Doxycycline was used at 1 mg/ml final concentration (ThermoFischer J63805.06). JS1 cells (kind gift from Youngmin Lee, PhD (VUMC)) were maintained in high glucose DMEM (Gibco 11965-092) supplemented with 2% FBS and penicillin/streptomycin.

Generation of stable cell lines with inducible ZNF469 constructs

For the generation of stable transgenic LX-2 cell lines, 500,000 cells per well were seeded in a 6-well plate and were transfected with Lipofectamine 3000 according to the manufacturer's protocol (Invitrogen L3000015). For the transfections, 2.1 mg of the plasmid containing the inducible ZNF469 constructs were combined with 400 ng of the PiggyBac helper plasmid *(1)*. Transfected cells were left for 3 days and subsequently selected with 250 mg/mL Geneticin (Gibco 10131035) for 2 weeks. Medium and Geneticin were replenished every 2-3 days.

siRNA knock down

JS1 mouse stellate cell line was transfected with a pool of 4 siRNAs targeting Zfp469 (ON-TARGETplus Mouse Zfp469 siRNA, Horizon Discovery, catalogue #L-061504-01-0005) using electroporation (program #8) on the NEON transfection system. A negative control was also included ON-TARGETplus Non-targeting Control Pool (Horizon Discovery catalogue # D-001810-10-05). After electroporation, cells were plated at 10,000 cells per well in a 96-well plate. RNA was extracted for one-step RTqPCR gene expression analysis 24 h after plating.

Inducible ZNF469 constructs

Human full length ZNF469 coding sequences as well as a version carrying a deletion was cloned into a doxycycline inducible PiggyBac expression vector described previously (1). The sequences of the corresponding constructs piHA-ZNF469_FL_PB and piHA-ZNF469_dZF_PB were submitted to GenBank (submission IDs: PP266319 and PP266320, respectively).

Molecular biology design and analysis software

Geneious Prime was used for construct design and protein sequence analysis. PSORT was used for identification of nuclear localization signals (PSORT: Protein Subcellular Localization Prediction Tool (genscript.com))

RNA extraction and quantification

RNA from human cell cultures was extracted from 6 well plates using RNeasy Plus- or RNeasy- micro kit (Qiagen, 74034 or 74004). The plates were washed once with PBS, 350 or 500 ml RLT Plus buffer was added to each well and the plates were kept at –80 °C until extraction. Qiagen's standard protocol including a DNase treatment was followed and the total RNA was finally eluted into 20 ml RNase-free water. RNA quality was assessed using a Tapestation (Agilent) and quantity measured using the Qubit RNA BR assay (Thermo Fisher

Scientific, Q10210). For qPCR, 2 ml RNA were used in each PCR reaction using KAPA SYBR FAST One kit (KK4651) and run on a QuantStudio 7 Flex System (Applied biosystems).

For LX-2 cells total RNA was reverse transcribed into complementary DNA with the SuperScript VILO cDNA synthesis kit (Invitrogen, 11754050). Quantification of the complementary DNA template was performed by real-time PCR using SYBR green fluorescence on a Bio-Rad CFX96 thermal cycler. Expression was normalized to that of 18S as an internal control. We used the 2-ΔΔCt method to determine the relative RNA levels.

Total RNA from frozen liver tissue (30 mg) was isolated using the Qiagen RNeasy Mini kit (Germantown, MD), per manufacturer's instructions. RNA quality was assessed using the 2100 Bioanalyzer (Agilent). For JS1 cells, RNA was extracted using 40 microliters of Bio-Rad RNA sample preparation reagent (Catalogue #1708898) and frozen for subsequent qPCR with BioRad one-step SYBR green RT-qPCR reagent (Catalogue #1725150). One ml of RNA was used in each PCR reaction. The primers used in this study are listed in Supplemental Table 11.

RNA-sequencing

NGS libraries were prepared with the Illumina TruSeq Stranded mRNA Sample Preparation kit (Illumina, #20020595) according to the manufacturer's instructions (Illumina TruSeq Stranded mRNA Reference Guide, # 1000000040498 v00, October 2017), using 400 ng of total RNA as input and applying 12 cycles of PCR at the final enrichment step. The produced NGS libraries were quality controlled using the D1000 ScreenTape and D1000 reagents (Agilent, #5067-5582 and 5067-5583) on the Agilent 2200 TapeStation system. Quantification of the libraries was performed using the Qubit dsDNA HS Assay Kit (Thermo Fisher, #Q32854) on the Biotek FLx800 Fluorescence plate reader. Paired-End 51 bp data were produced using the Illumina NovaSeq 6000 system, generating 26 million reads per sample on average.

The sequencing data were base called with Illumina's Real-Time Analysis (RTA v3.4.4) software and processed into FASTQ file format, containing the sequence data, with the bcl2fastq2-v2.20.0.422 tool.

RNA-sequencing analysis

We used the exon quantification pipeline (version 2.5, (2)) with STAR (version 2.7.3a, (3)) to align the reads against the human genome reference from Ensembl version 98 (4) and used PISCES (GitHub - Novartis/pisces) to quantify gene expression.

For liver samples data principal component analysis was performed on the top 5% genes with the highest median absolute deviation using the prcomp R function. Differential gene expression between disease stages (NOR vs MASH_F2/3) was determined using DESeq2 (v1.38.3) with sex as covariate. Genes were defined to be differentially expressed if FDR <= 0.01 and |log2FC| > 0.5. All differential gene-based enrichment analysis was performed with the enricher function from ClusterProfiler (v4.6.2) R package. Cell type enrichment analysis for these differential disease genes was performed with marker gene sets from Aizarani et al. (5). The HSC gene set was defined by the overlap between MASH_F2/3 genes and the enriched HSC markers from Aizarani et al. Gene ontology analysis for the same differential genes was based on the Hallmark, Wikipathways, Reactome and NABA gene sets from MSigDB (v7.5.1).

For HSCs with CRISPR perturbation differential gene expression and gene set enrichment analysis was performed as described in section above. Here we compared CRISPR knockouts with non-targeting control (hNTO). For the gene set enrichment analysis we used the gene ontology biological processes gene sets from MSigDB (v7.5.1).

The CRISPR editing analysis was performed by first matching back the location of gRNAs to the human genome using the vmatchPattern function from the R-package Biostrings (v2.64.1). Genotyping of the gRNA locations was performed using the pileup function from Rsamtools (v2.14.0) with following parameters: distinguish_strands = FALSE, distinguish_nucleotides = TRUE, ignore_query_Ns = TRUE, include_deletions = TRUE, include_insertions = TRUE.

Proteomics data analysis

Triplicate samples of hNTO and *ZNF469* cells were analyzed using multiplexed TMT-based proteomics methodology essentially as described (6). Briefly, cell pellets were extracted and digested to peptides using the iST-NHS Sample Preparation Kit (PreOmics, #P.O.00083) according to the kit protocol. Equal amounts of the purified peptide mixtures were labeled with TMT 18-plex reagents (Thermo Fisher Scientific), mixed after confirming complete labeling, and fractionated into 24 concatenated fractions using high pH reverse phase HPLC. These 24 fractions were then analyzed by liquid chromatography-mass spectrometry as described (Ferretti et al.). Raw data were analysed using Proteome Discoverer 3.0 (Thermo Fisher Scientific) and further processed using in-house Python scripts for quantification and LIMMA-based statistical analysis. Quantification is based on median-normalized summed intensities of unique peptides for each protein.

CUT&RUN chromatin profiling

CUT&RUN was carried out as described previously with the following modifications (7).

For CUT&RUN on liver tissues flash-frozen pieces of human liver biopsies were weighted (around 1 g per sample) and transferred to pre-cooled tissue TUBES TT1 (Covaris, PN 520128). Tissues were pulverized with the CP02 cryoPREP automated Dry Pulverizer from Covaris by executing one pulse at intensity level 2 followed immediately by a second pulse at intensity level 4. While constantly kept on dry ice, around 5 mg of tissue powder per donor were transferred into BSA-pre-coated and pre-cooled 1.5 mL Eppendorf tubes. Tissues were washed with 1 mL of cold DPBS and centrifuged for 5 min at 1000 x g (4 °C). Tissues were fixed for 5 min at room temperature (RT) with 1% fresh formaldehyde (Pierce 16% Formaldehyde Methanol Free, 28906) diluted in PBS under gentle rotation. The fixation was quenched with 1:10 vol of 1.25 M glycine solution for 5 min with gentle rotation. Samples were washed again with DPBS and resuspended in 1 mL of NE Buffer (20 mM HEPES pH 7.5, 10 mM KCl, 0.5 mM Spermidine, 0.1 % Triton X-100, 20 % Glycerol, cOmplete EDTA-free Protease Inhibitor Cocktail, 4693132001). Samples were transferred into Lysing Matrix D 2 mL tubes containing 1.4 mm ceramic beads (MP Biomedicals, 116913050-CF) and treated for 10 sec at 5000 rpm with the Precellys 24 Tissue Homogenizer from Bertin Technologies. Cell suspensions were quickly

placed on ice for 5 min and filtered through a 70 µm cell strainer, then transferred into new BSA-pre-coated 1.5 mL Eppendorf tubes and centrifuged for 3 min at 500 x g. Finally, pellets were resuspended in 100 µL of Wash 1 Buffer (20 mM HEPES pH 7.5, 150 mM NaCl, 0.5 mM Spermidine, cOmplete EDTA-free Protease Inhibitor Cocktail, (4693132001) and further processed as described below.

For cell cultures cells were detached with 0.25% Trypsin-EDTA (Gibco, 25200056) for 5 min at 37°C, centrifuged at 300 x g for 3 min, washed once with PBS and counted with Countess cell counter (Thermo Fisher). The fixation with formaldehyde and the quenching were done as described above. Cells were washed with PBS twice, then re-suspended in PBS and transferred into a final volume of 500 mL in 1.5 mL Eppendorf tubes pre-coated overnight with 1% BSA. Aliquots of 2 million-, or 200,000-cells were used for the ZNF469, or for the H3K27ac profiling, respectively, and processed as described below.

For all sample sources processed as described above, Activated BioMag® Plus Concanavalin A magnetic beads (Bangs Laboratories, BP531) were added to the cell suspension (40 µL for ZNF469 or 10 µL for H3K27ac) and incubated for 10 min with gentle rotation. After magnetic separation, cells were resuspended in 200 µL of DIG-wash EDTA Buffer (20 mM HEPES pH 7.5, 150 mM NaCl, 0.5 mM Spermidine, 0.02% Digitonin, 2 mM EDTA, cOmplete EDTA-free Protease Inhibitor Cocktail) with the primary antibody for 2 h with gentle rotation. Primary antibodies used were rabbit polyclonal ZNF469 (Sigma, HPA069784), polyclonal H3K27ac (Abcam, ab4729). Cells were washed twice with 1 mL of DIG-wash EDTA buffer and incubated for 1 h at 4 °C with gentle rotation in 200 µL of DIG-wash EDTA Buffer with CUTANA pAG-MNase (protein A- protein G-Micrococcal Nuclease fusion, Epicypher, 15-1016). Subsequently, the cells were washed twice with 1 mL of DIG-wash EDTA Buffer and once with 1 mL of DIG-wash Buffer without EDTA. For the pAG-MNase digestion, the cells were re-suspended in 100 µL of cold DIG-wash Buffer without EDTA, then $CaCl_2$ was added to a final concentration of 2 mM to activate the MNase. Samples were incubated for 30 min at 0°C in an aluminum block. Digestion was terminated by adding 100 µL of 2X Stop Buffer (400 mM NaCl, 20 mM EDTA, 4 mM EGTA, 0.02% Digitonin, 100 µg/mL RNase A, 50 µg/mL Glycogen) to the samples. Samples were incubated for 30 min at 37 °C without shaking. Supernatants were collected and treated with SDS (1% final concentration) and 200 µg/mL Proteinase K for minimum 4 h at

65°C. DNA was extracted by phenol-chloroform for the ZNF469 profiling to keep the small DNA fragments and with the DNA Clean & Concentrator-5 kit (Zymo Research, D4004) for the H3K27ac profiling. Purified DNA was eluted with the EB buffer (Qiagen, 19086) and transferred in 1.5 mL DNA LoBind tubes (Eppendorf, 30108051).

For library preparation, the NEBNext Ultra II DNA Library Prep kit for Illumina (New England Biolabs, E7645L) was used as described previously (8, 9). The end repair step was adapted for each target: 30 min at 20°C and then 1 h at 50°C for ZNF469; 30 min at 20°C and then 30 min at 65°C for H3K27ac. The NEB adaptor was diluted at 1:15. The NEBNext Multiplex Oligos for Illumina SET2 (New England Biolabs, E7500L) were used for the indexing. The PCR reaction was adapted for each target: 9 cycles and annealing / extension for 10 sec at 65°C for ZNF469; 8 cycles and annealing / extension for 75 sec at 65 °C for H3K27ac. Libraries were purified using Agencourt AMPure XP beads (Beckman Coulter, A63881) and the concentrations and profiles were checked on TapeStation with a D1000 Screen Tape. Libraries were sequenced using NovaSeq6000 paired-end mode 2 x 51 bp.

CUT&RUN data analysis

For the H3K27ac liver cohort principal component analysis was performed on the top 5% peaks with the highest median absolute deviation using the prcomp R function. Like RNA-seq, differential H3K27ac signal between NOR and MASH_F2/3 was tested using DESeq2 (v1.38.3) with sex as covariate. We defined differential H3K27ac regions as FDR <= 0.01 and |log2FC| > 0.5. Metaprofiles and IGV tracks of CUT&RUN and other chromatin signals were computed using a custom script which is based on the ScoreMatrix function from genomation (v1.19.1) R-package. Cell type specific scATAC-seq peaks were obtained from Zhang et al (10). NOR and MASH specific differential regions overlapped with these peaks. Resulting confusion matrices were used to test for enrichment with two-sided Fisher’s exact tests.

For H3K27ac in HSCs, differential H3K27ac between hNTO and ZNF469 KO was determined as described in the previous section. Peaks were identified as differentially acylated if FDR <= 0.01 and |log2FC| > 0.5.

HSC ZNF469 KO RNA-seq and H3K27ac CUT&RUN were integrated by comparing the log2FC of gene expression and H3K27ac at the promoter of a gene or distal intergenic peaks were assigned to genes using the capture Micro-C interactions.

For ZNF469 in HSCs, ZNF469 binding was determined against a knockout control using DESeq2 (v1.38.3) to compare ZNF469 CUT&RUN hNTO against ZNF469 KO. Since we expect genome-wide changes in TF binding, we computed size factors based on *E. coli* spike in reads as previously described. ZNF469 binding sites were defined using following thresholds FDR <= 0.05 and |log2FC| > 0.5.

ChIPseeker (v1.34.1) was used to annotate the binding sites with their genomic locations. Enrichment of the ZNF469 binding sites was determined by comparing their genomic location with the ones of non-significant bound sites using a one-sided Fisher's exact test.

Micro-C and promoter capture

To generate a genomic promoter interaction map in human primary hepatic stellate cells (HSC190131) two replicas of 1-1 million cells were fixed with disuccinimidyl-glutarate and processed with the Dovetail Micro-C Kit (21006) following a promoter capture step (Dovetail Human Pan Promoter Enrichment Kit, 25013) according to the manufacturer's manual. For two replicates 494 million fragments were sequenced on the Illumina NovaSeq 6000 system. The above reagents as well as Dovetail Library Module for Illumina (25004), Dovetail Dual Index Primer Set #1 for Illumina (25010), and the Promoter Panel Informatics Analysis service (8015) were from Dovetail Genomics.

Micro-C and promoter capture analysis

Capture Micro-C preprocessing was performed as outlined at https://dovetail-capture.readthedocs.io/. Briefly, paired-end reads were aligned to hg38 using bwa meme (v2.2.1) with following parameters: -5SP -T0. Resulting alignment was searched for valid ligation events using the parse function from the pairtools pipeline (v1.0.2) with following parameters: --min-mapq 40 –walks-policy 5unique –maxinter-align-gap 30. PCR

duplicates were removed using pairtools dedup and bam files for downstream analysis were generated using samtools (v1.13). Chromatin-chromatin interactions calling was performed using CHiCAGO (v1.26.0). Therefore, bait fragment (BF) and other end fragments (OEF) maps were generated at a 5kb resolution. These maps were used to compute the coverage from the bam files using the bam2chicago.sh script. Resulting compatible input files were used for interaction calling with CHiCAGO. Interactions with a score higher than 5 were considered significant. Bait plots were plotted based on CHiCAGO generated statistics with custom ggplot2 (v3.4.4) based R code.

Analysis to identify transcription factor candidates

To identify candidate TFs different methods were used with the appropriate input data as described below.

ANANSE (11): First, we identified nucleosome free regions (NFRs) per patient using HisTrader (github.com/SvenBaileyLab/HisTrader) with default parameters and H3K27ac CUT&RUN data as input. We combined these NFRs per disease stage (NOR, MASH_F2/3) by merging the bed files using BEDtools (v2.27.1). TF binding to disease stage NFRs were predicted using ANANSE binding (default settings) in combination with the merged locations and H3K27ac CUT&RUN signals. Next, gene regulatory networks per disease stage were reconstructed using these TF binding predictions in combination with gene expression measurements from RNA-seq as input for the ANANSE network function. Finally, TFs were prioritized using ANANSE influence (default settings) with the DESeq2 differential genes between NOR and MASH_F2/3, the gene regulatory networks (GRNs) for NOR AND MASH as input.

CRCmapper (12): We identified super-enhancers from H3K27ac CUT&RUN for each patient using ROSE2 (bitbucket.org/young_computation/rose) with default settings. These were used to construct Core Regulatory Circuitries (CRCs) using CRCmapper (github.com/linlabcode/CRC) in combination with NFRs as subpeaks (see ANANSE) and actively expressed genes as defined by matched RNA-seq (TPM > 1). Resulting patient level CRCs were used as input to compute various network statistics (in degree, out degree, total degree,

betweenness, alpha centrality and eigenvector) using the R-package igraph (v1.3.4). We compared the out degree network statistics between NOR and MASH_F2/3 using Wilcoxon rank sum tests.

DoRothEA (v1.10) (13): Curated GRNs for Homo sapiens were queried via the DoRothEA R-package. To compute a TF activity score per patient from RNA-seq, we used these GRNs in combination with the TPM matrix as input for the run_ulm function from DecouplR (v2.4; minsize = 5). Resulting TF activity scores per patient were summarized by comparing NOR vs MASH_F2/3 using Wilcoxon rank sum tests.

Homer (v4.11) (14): The motif consensus library from Lambert et al was used for the motif enrichment with HOMER. The analysis itself was performed using the function findMotifsGenome with MASH_F2/3 as foreground and NOR differential regulatory region as background sequences.

MonaLisa (v1.2) (15*)*: Motif enrichment with MonaLisa was performed using the motif consensus library from Lambert et al. Motif counts were obtained by matching the Lamber et al PWMs to the H3K27ac consensus peaks using the matchMotif function from motifmatchr. We computed GC and CpG observed/expected ratios were computed as additional features. The motif counts and ratios were used to model the H3K27ac log2(FC) between NOR and MASH_F2/3 using the randLassoStabSel function from MonaLisa (cutoff = 0.8).

RcisTarget (v1.18.2) (16): We used RcisTarget to compute the motif enrichment for MASH_F2/3 differential regions. Coordinates of differential regions were first converted from hg38 to hg19 using liftOver (v1.58). Region based motif enrichment was performed using the hg19 ranking database and NOR peaks as background. The analysis was performed by running the cisTarget function from RcisTarget.

sgRNA stocks preparation

For the arrayed CRISPR screen, crRNAs were either cherrypicked from Horizon's Human Edit-R Drug targets (GC-004650-05) or Human Druggable Subset (GC-004670-05) synthetic crRNA libraries or were purchased

from IDT. Individual crRNAs from the Horizon libraries (0.5nmol/well) were resuspended into 35 ml 10mM Tris pH7,4 to generate a 15 mM solution using a Multidrop Combi reagent dispenser (ThermoFisher Scientific), placed on an orbital shaker for a few minutes and incubated at RT for about 1h. They were cherrypicked from the original library plates and 4 crRNAs per target were pooled (4 x10 ml) and re-arrayed using a Biomek-FX automated pipetting system (Beckman Coulter). Edit-R CRISPR-Cas9 Synthetic tracrRNA (Horizon Discovery Biosciences, U-002005-20) was resuspended and diluted to 15 mM in 10 mM Tris pH 7.4 and 40 ml were then added to the crRNAs using a Multidrop Combi dispenser and incubated at RT for 20 min. The guides were then transferred to stock plates using a CyBio SELMA 96/60ul semi-automated pipette (Analytik Jena) according to the final layout and kept at -20°C (final concentration of 7.5 mM). For all targets that were not available from the Horizon libraries, crRNAs were purchased from IDT as a pool of 4 x 2 nmol crRNAs (Alt-R CRISPR-Cas9 crRNA, sequences according to T.spiezzo library, (17). They were resuspended in 10mM Tris pH 7.4 to obtain 100 mM stocks, further diluted to 15 mM and incubated for 20min at RT with 15 mM Edit-R CRISPR-Cas9 Synthetic tracrRNA. They were finally transferred to the stock plates using a multichannel pipette according to the final layout and kept at -20°C. For simplicity the complexed crRNA-tracrRNA molecules will be referred to as ”sgRNA” in the manuscript.

RNP preparation and delivery

For the arrayed CRISPR screen, 7.5 mM sgRNA stock plates were thawed and 5.76 ml of each guide was transferred to a 96 well conical bottom plate (Nunc, 249935) using a CyBio SELMA. Control sgRNAs were added manually. Alt-R S.p. Cas9 Nuclease V3 (IDT, 1081059) was diluted to 15 uM in 10 mM Tris-HCl pH 7.4 and 2.4 ml were dispensed on top of the sgRNAs using a Mantis microfluidic liquid dispenser (Formulatrix). The RNP complexes were incubated at RT for 15min and then kept at 4°C until use. During this time, human HSCs were detached and resuspended in P3 buffer (Lonza, P3 primary cell 384-well nucleofector kit) containing Alt-R Cas9 Electroporation Enhancer (IDT, 1075916). 15.84 ml of cells were then pipetted onto the prepared RNP complexes using a multichannel pipet (36 pmol RNP for 30,000 cells)

and 20 ml of that mix was transferred to a Lonza 384-well nucleofector plate (30 pmol RNP and enhancer for 25,000 cells per condition) and electroporated using program CA-137 on a 384-well Nucleofector system. 40 ml fresh medium were added to the electroporation plate and 2 ml cells were transferred into prefilled and prewarmed collagen-coated 96 well plates (Biocoat Collagen I 96-well clear flat bottom TC-treated microplates, Corning, 356698) in multiple replicates using a Cybio SELMA (final seeding density of 800 cells/well). The plates were incubated at 37°C and a medium change was performed 4 days after electroporation. The plates were then processed as detailed in the other sections.

Conditions were adapted and scaled up for each of the validation experiments according to the desired final cell amounts. RNP ratios per cell were kept constant and ranged from 30 pmol RNP for 25,000 cells to 480 pmol RNP for 400,000 cells. Seeding densities were also scaled up to mimic the conditions of the screen and fresh crRNAs were purchased from IDT for experiments requiring more concentrated RNPs.

For CRISPR targeting of *Zfp469* in JS1 cells, RNPs were generated using two crRNAs per region of the gene with one gRNA pair targeting the proximal region and another pair targeting the zinc finger region (base pairs 9538-9661) of *Zfp469* open reading frame. 3 ml of crRNA and tracrRNA (stocks 100 mM) were annealed together at 95°C in 4 ml of R buffer (NEON transfection system, Thermo Fisher, Cat. #MK10096). After cooling for 10 mi at RT, 1.5 ml of Cas9 nuclease (spCas9, IDT #1081058, stock 10 mg/mL) was incubated with crRNA/tracrRNA for 30 min at 20°C. During this incubation, cells were collected and counted for electroporation at a concentration of 1.25e6 cells per 110 ml of R buffer. Electroporation was performed per manufacturer protocol (NEON) in 3 mL buffer E2 per tube with 100 ml tips after mixing RNP with cells. Protocol used for electroporation: Pulse Voltage = 1300, Pulse Width = 20, Pulse Number = 2.

Immunofluorescence staining

All the steps were done at RT. For imaging cells in micro-well plates (Greiner Bio-One 384 Collagen type I CELLCOAT 384 well, 781956) cells were fixed directly by adding paraformaldehyde (PFA) (Electron Microscopy Sciences, 15714S) to the medium (final concentration 4%) for 15 min on a shaking platform.

Cells were washed (80 ml) 3 times with PBS by using the Washer Dispenser EL 406 from Biotek. Cells were permeabilized with Blocking Buffer (PBS pH 7.5, 2 % BSA, 0.1% Triton X-100) for 45 min, followed by 3 washes with Wash 2 Buffer (PBS pH 7.5, 0.1% Triton X-100). Cells were then incubated with the primary antibody in Blocking Buffer for a minimum of 3 h. For COL1A1 staining the monoclonal mouse anti human COL1A1 antibody (Developmental Studies Hybridoma Bank, M-38, lot 5/13/2021) was used at a dilution of 1:1500. After 3 washes with Wash 2 Buffer, the cells were incubated for 1 h with the secondary antibody (goat anti-mouse Alexa Fluor 488, Invitrogen, A11029) diluted at 1:1000 in the Blocking Buffer supplemented with Hoechst diluted at 1:10,000. Finally, cells were washed 3 times with the Wash 2 Buffer (PBS pH 7.5, 0.1% Triton X-100) and stored with aluminum sealing in PBS at 4°C until imaging. For LX-2, cells plated in 96-well plates (Greiner 96-well microplates, µclear®, 655097) were fixed in 100 ml PBS + 4% PFA for 15 min at RT and washed 3 times with 150 ml of PBS. All the subsequent steps were done as described above. The anti-ZNF469 antibody (Sigma, A31572, lot R98565) was diluted 1:400. Secondary antibody (donkey anti-rabbit Alexa Fluor 555, Invitrogen, A31572) dilution was 1:1000. DAPI (Sigma D9542, lot 175158) was used at a final concentration of 1 ng/mL.

Image acquisition, processing and quantification

For the CRISPR screen the immunofluorescent images were captured with the confocal dual spinning disk Cell Voyager CV7000 and quantified with High Content Analysis Software CellPathFinder (Yokogawa). Confocal images of LX-2 cells were captured with ZEN 3.2 on the Zeiss Axio Observer Z1 microscope with 63x magnification (Plan-Apochromat 63x/1.40 Oil DIC M27) and images were processed with Fiji (18). Confocal images from RNAscope experiments were collected on a Zeiss LSM880 using a 63x/1.40 Plan-APOCHROMAT OIL objective. MERSCOPE images were visualized with MERSCOPE visualizer v2.

RNAscope

For RNAscope of mouse livers, mice were perfused with 4% PFA for 10 min and tissues were harvested and put in 4% PFA for 24 h at 4°C. After fixation, samples were paraffin embedded and sectioned (i.e. FFPE). FFPE liver tissues were subsequently stained using the RNAscope Multiplex Fluorescent Detection Reagents_v2 (ACDBio, catalogue #323110). Target retrieval was performed for 30 min at 100°C. Protease treatment used was Protease Plus for 30 min at 40°C. RNAscope probes used were Mm-Col1a1-C3 (ACD #319371-C3), Mm-Acta2 (ACD #319531), Mm-Zfp469-C2 (ACD #1142291-C2, detection by Opal Fluorophores (Akoya Biosciences - Opal 520 #FP1487001KT, Opal 570 #FP1488001KT Opal 690, #FP1497001KT).

MERSCOPE sample processing

Five-micron thick sections of FFPE human liver specimens were prepared and placed in groups of four on round glass slides provided by Vizgen Corp. (MERSCOPE slide part number 20400101). One mL of a fiducial premix dilution (1:500 in 1XPBS) was added on top of each MERSCOPE slide. Slides were dried at 55°C for 15 min then 2 hrs at RT before overnight shipment on dry ice to European Spatial Biology Center (Leuven, Belgium) for routine processing.

MERSCOPE analysis

For the cell-segmentation task, we used Vizgen Post-processing Tool (VPT, v1.0.2) with python (v3.9.6). Cell segmentation was performed on DAPI, polyT and Cellbound2 staining from the z1-plane using the "segmentation family" Cellpose (19) in cyto2/2D mode with a cell diameter of 100 mM. For the transcript-to-cell assignment, we used maximal projectzion from the different z-planes to the respective cells determined in layer z1. With VPT we obtained the cell-metadata and the cell-by-gene matrix files. Cells were filtered for at least 30 different detectable transcripts and used for downstream integration with liver scRNA-seq.

We obtained pre-processed and annotated liver scRNA-seq from Wang *et al* (20). For the integration, we filtered the scRNA-seq and MERSCOPE matrices for overlapping features (n = 297), merged both matrices and split the matrix by donor again. The resulting matrix list was used for downstream analysis using Seurat (v5.0.1). Briefly, counts per donor were normalized using NormalizeData (default), followed by dimensionality reduction via PCA on the scaled variable features. Then the datasets were integrated based on the PCA per donor using Harmony (21).

We chose the first 10 Harmony dimensions to perform the UMAP embedding (RunUMAP; default) and shared nearest-neighbor graph construction (FindNeighbors; default). Cells were clustered using FindClusters with resolution = 0.3. The resulting clusters were annotated using the pre-existing cell type annotation from the scRNA-seq dataset, based on the most enriched cell type in each cluster.

To determine the co-expression of *ZNF469* with all other transcripts, we binarized the transcript expression and performed one-sided Fisher's exact tests to test the enrichment of other transcripts in *ZNF469* positive cells. This was done on an individual patient level and over the combined analysis of all samples.

References

1. Rose N, et al. Receptor clustering and pathogenic complement activation in myasthenia gravis depend on synergy between antibodies with multiple subunit specificities. *Acta Neuropathol*. 2022;144(5):1005–1025.

2. Schuierer S, Roma G. The exon quantification pipeline (EQP): a comprehensive approach to the quantification of gene, exon and junction expression from RNA-seq data. *Nucleic Acids Res*. 2016;44(16):e132–e132.

3. Dobin A, et al. STAR: ultrafast universal RNA-seq aligner. *Bioinformatics*. 2013;29(1):15–21.

4. Cunningham F, et al. Ensembl 2015. *Nucleic Acids Res*. 2015;43(D1):D662–D669.

5. Aizarani N, et al. A human liver cell atlas reveals heterogeneity and epithelial progenitors. *Nature*. 2019;572(7768):199–204.

6. Ferretti S, et al. Discovery of WRN inhibitor HRO761 with synthetic lethality in MSI cancers. *Nature*. 2024;629(8011):443–449.

7. Meers MP, et al. Improved CUT&RUN chromatin profiling tools. *Elife*. 2019;8. https://doi.org/10.7554/eLife.46314.

8. Liu N, et al. Direct Promoter Repression by BCL11A Controls the Fetal to Adult Hemoglobin Switch. *Cell*. 2018;173(2):430-442.e17.

9. Liu N. Library Prep for CUT&RUN [Internet]. *https://dx.doi.org/1017504/protocols.io.wvgfe3w*. 2019.

10. Zhang K, et al. A single-cell atlas of chromatin accessibility in the human genome. *Cell*. 2021;184(24):5985-6001.e19.

11. Xu Q, et al. ANANSE: an enhancer network-based computational approach for predicting key transcription factors in cell fate determination. *Nucleic Acids Res*. 2021;49(14). https://doi.org/10.1093/nar/gkab598.

12. Saint-André V, et al. Models of human core transcriptional regulatory circuitries. *Genome Res*. 2016;26(3):385–396.

13. Garcia-Alonso L, et al. Benchmark and integration of resources for the estimation of human transcription factor activities. *Genome Res*. 2019;29(8). https://doi.org/10.1101/gr.240663.118.

14. Heinz S, et al. Simple combinations of lineage-determining transcription factors prime cis-regulatory elements required for macrophage and B cell identities. *Mol Cell*. 2010;38(4). https://doi.org/10.1016/j.molcel.2010.05.004.

15. Machlab D, et al. monaLisa: An R/Bioconductor package for identifying regulatory motifs. *Bioinformatics*. 2022;38(9):2624–2625.

16. Aibar S, et al. SCENIC: single-cell regulatory network inference and clustering. *Nat Methods*. 2017;14(11). https://doi.org/10.1038/nmeth.4463.

17. Yin J-A, et al. Arrayed CRISPR libraries for the genome-wide activation, deletion and silencing of human protein-coding genes. *Nat Biomed Eng*. 2024;9(1):127–148.

18. Schindelin J, et al. Fiji: an open-source platform for biological-image analysis. *Nat Methods*. 2012;9(7):676–682.

19. Stringer C, et al. Cellpose: a generalist algorithm for cellular segmentation. *Nat Methods*. 2021;18(1):100–106.

20. Wang Z-Y, et al. Single-cell and bulk transcriptomics of the liver reveals potential targets of NASH with fibrosis. *Sci Rep*. 2021;11(1):19396.

21. Korsunsky I, et al. Fast, sensitive and accurate integration of single-cell data with Harmony. *Nat Methods*. 2019;16(12):1289–1296.

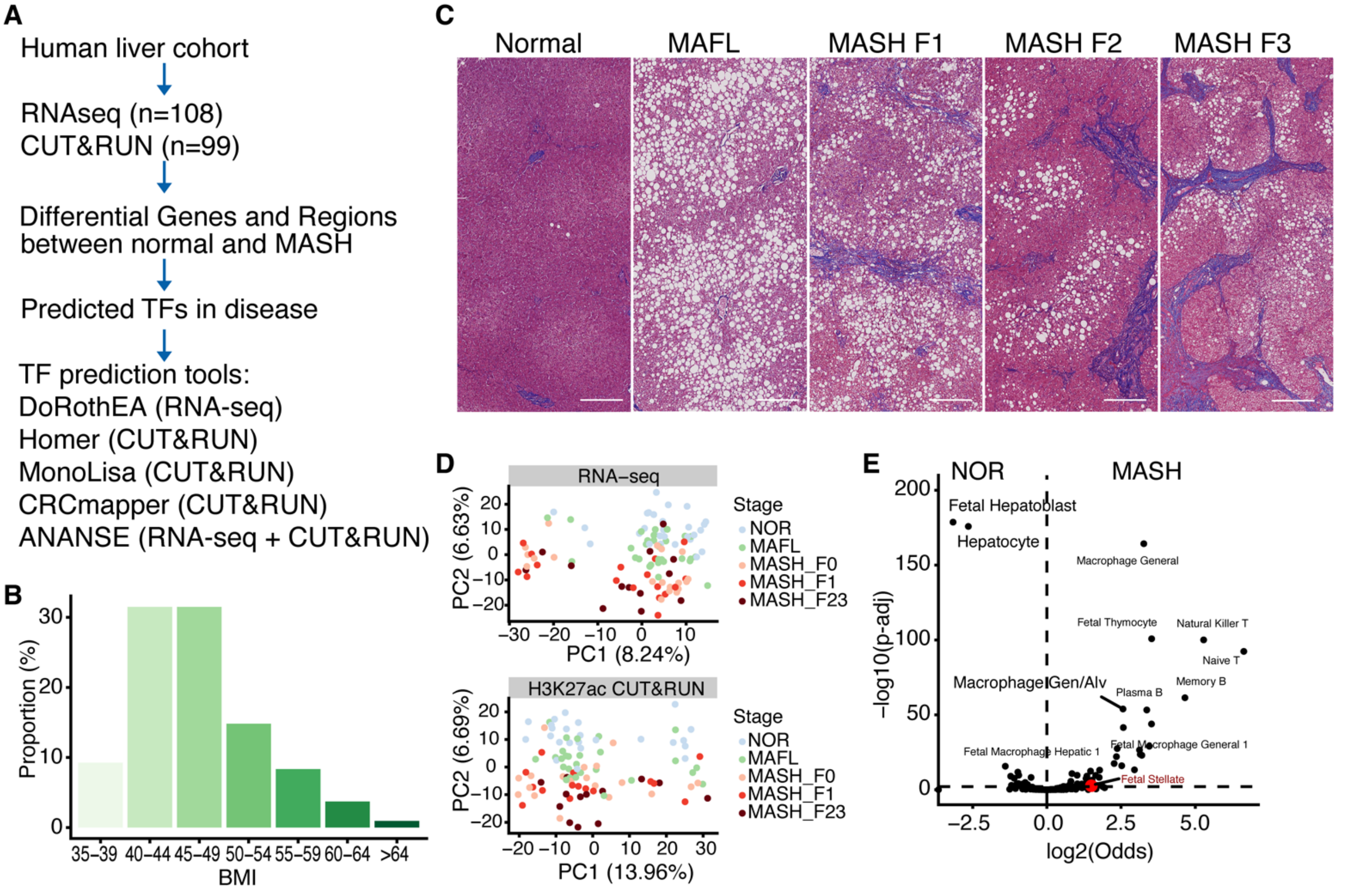

Supplemental Figure 1: **Integration of transcriptomics and cis-regulatory landscapes in human MASLD livers predicts activity of transcription factors involved in fibrosis**. (**A**) Schematic of multiomics data analysis workflow to identify potential transcription factors involved in disease progression. (**B**) Histogram of body-mass index (BMI) for the liver cohort (n=108). (**C**) Representative photomicrographs of Masson's Trichrome stained MASH liver biopsy samples demonstrating histology representative of normal (KUV229), MASL (ERH825), F1 fibrosis (OTZ834); (2) F2 fibrosis (YIU233) and (3) F3 fibrosis (DNM623). Scale bar = 100 microns. Slides were reviewed and scored by pathologists who were blinded to patient details and clinical status. (**D**) PCA of RNAseq and CUT&RUN colored by histopathology. (**E**) Volcano plot of scATAC-seq cell type specific peak enrichment in normal vs MASH.

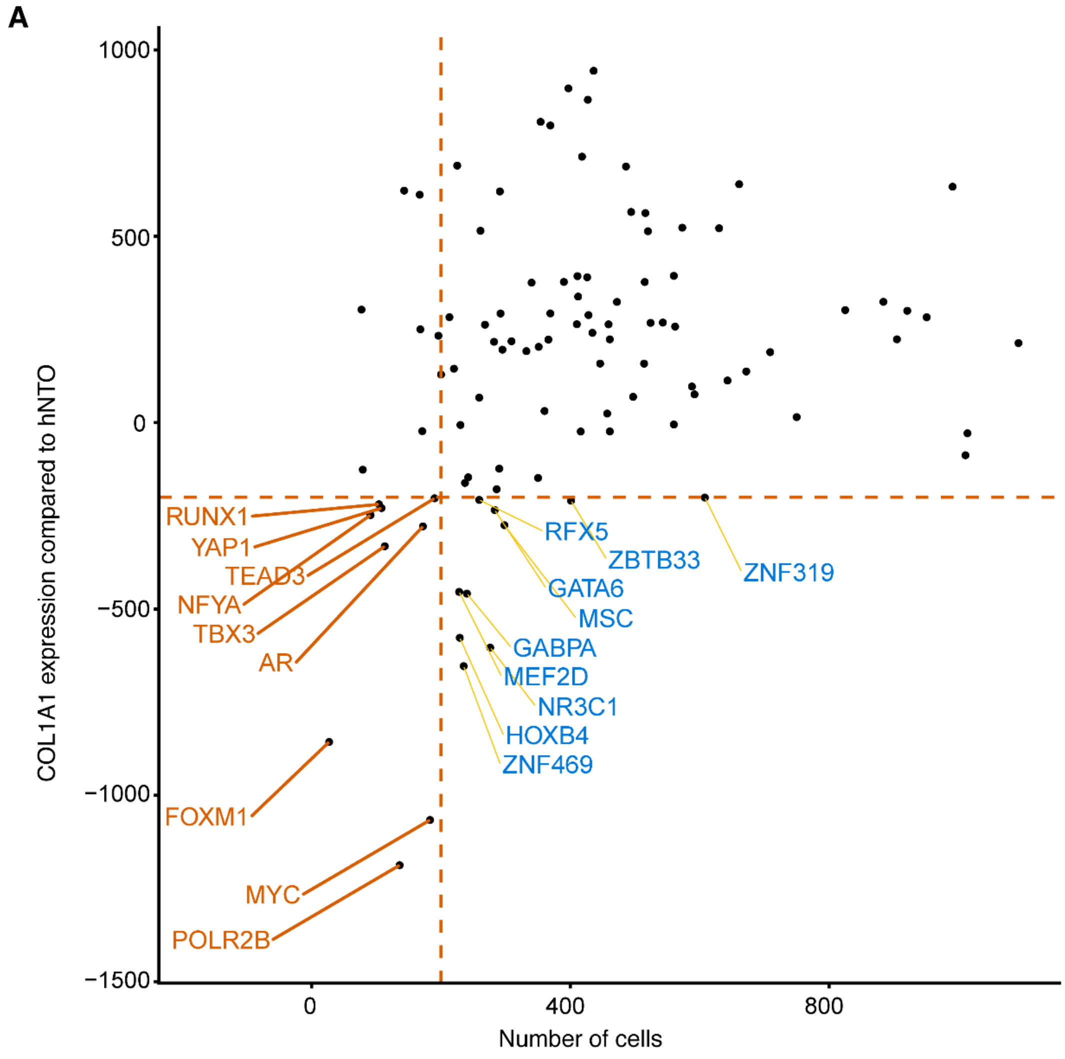

Supplemental Figure 2. CRISPR loss-of-function screen identifies transcriptional regulators of collagen production in primary human hepatic stellate cells. (A) Scatter plot showing cell count vs. COL1A1 immunofluorescence.

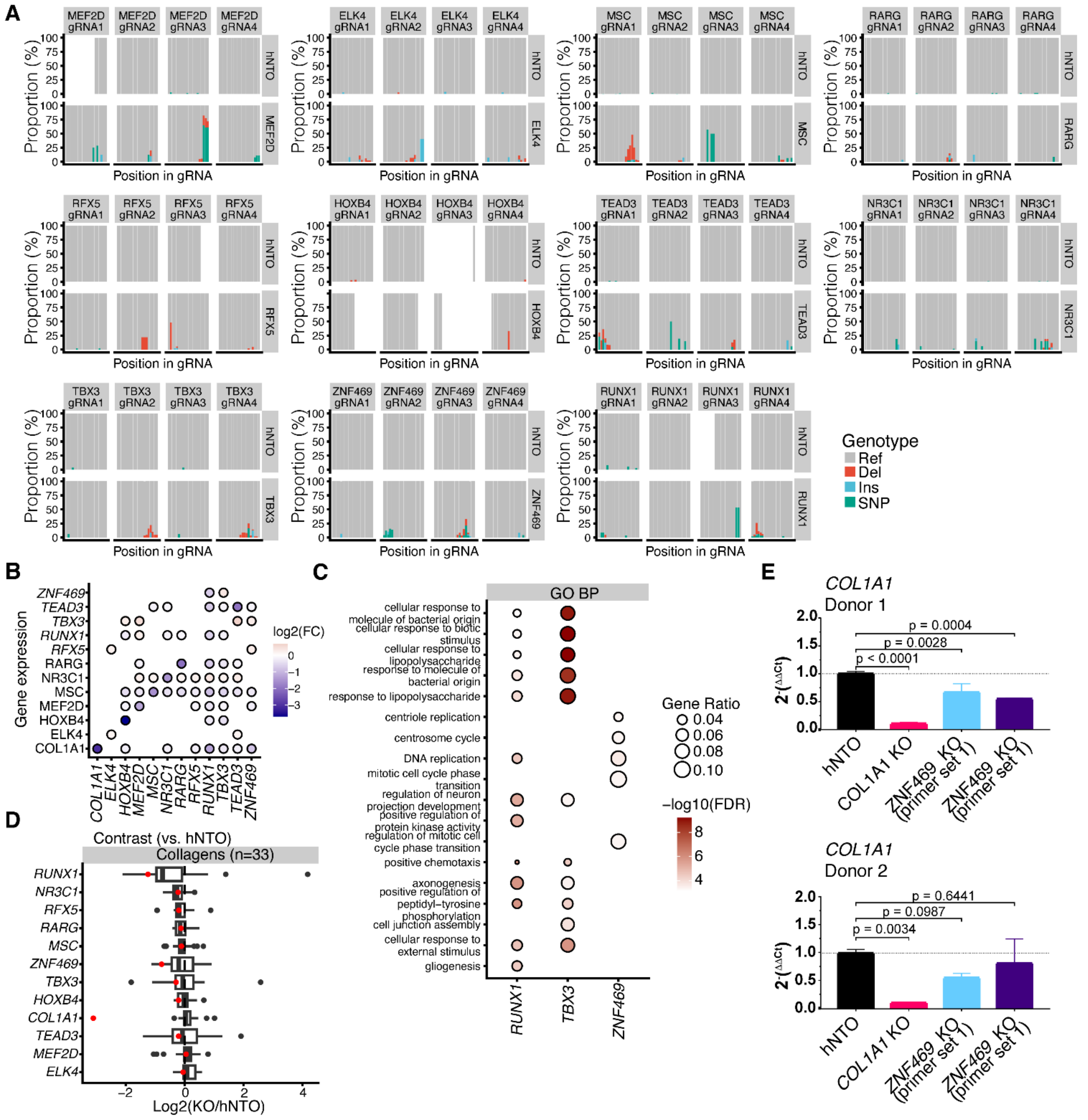

Supplemental Figure 3: *ZNF469* knockout alters collagen mRNA expression in HSCs. (A) RNA-seq indel plots shows editing confirmation for *MEF2D, ELK4, MSC, RARG, RFX5, HOXB4, TEAD3, NR3C1, TBX3, ZNF469, RUNX1*. (**B**) Dot plot of TF expression after KO of top hits from the CRISPR Screen. **(C)** Gene set enrichment of upregulated genes upon *ZNF469*, *RUNX1* or *TBX3* KO. **(D)** Gene Set enrichment of upregulated genes upon *ZNF469*, *RUNX1* or *TBX3* KO. **(E)** Bar plots of COL1A1 mRNA expression in primary HSCs after ZNF469 CRISPR KO (see Supplemental Table 5 for sgRNA sequences). In (E) Data normalized to housekeeper gene *RPL32*. Significance determined by one-way ANOVA and Dunnett's multiple comparison test. *p<0.05; **p<0.01,***p<0.001, ****p<0.0001.

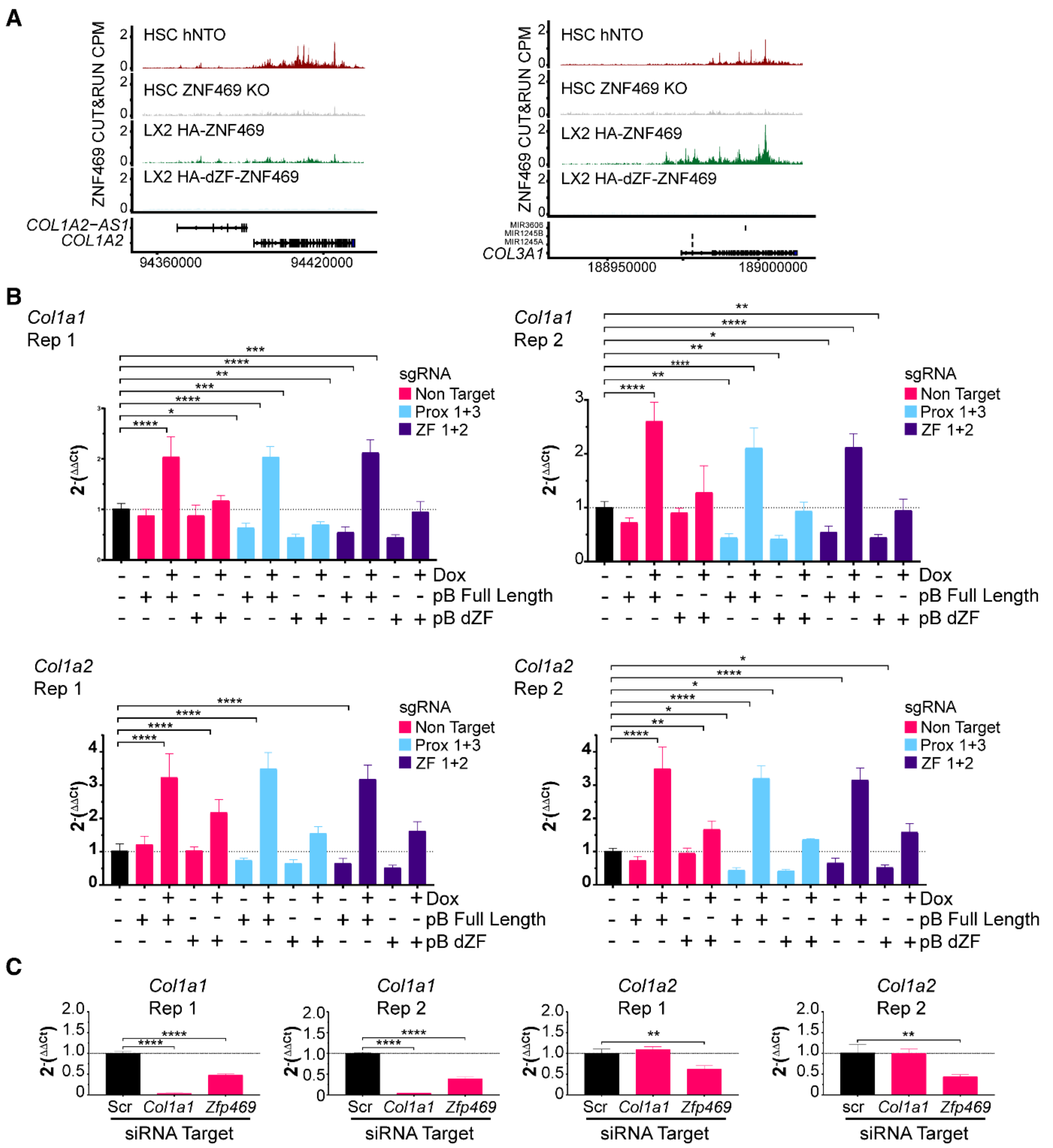

Supplemental Figure 4: ZNF469 binds at collagen gene regions and regulates collagen gene expression. (**A**) Genome browser tracks at the *COL1A2* and *COL3A1* locus of ZNF469 CUT&RUN signals using anti-ZNF469 antibody in CRISPR experiments in none-targeting **(NTO)** and *ZNF469* targeted human hepatic stellate cells and transgenic LX-2 cells with doxycycline inducible full length or deletion harboring *ZNF469* cDNA (**B**) Bar plots of *Col1a1* and *Col1a2* mRNA expression 7 days after *Zfp469* CRISPR KO in mouse JS1 cells and *ZFP469* rescue by overexpression of full length (FL) or ZF deleted (dZF) forms using the piggyBac system (pB), 24 h after transgene induction with doxycycline. One gRNA pair targeted the proximal region and another pair targeted the zinc finger region (base pairs 9538-9661) of *Zfp469* open reading frame. (**C**) Bar plots of *Col1a1* and *Col1a2* reduction following *Zfp469* knockdown after siRNA (24 h) in JS1 cells. p-values determined by one-way ANOVA and Dunnett's multiple comparison test. *$p<0.05$; **$p<0.01$,***$p<0.001$, ****$p<0.0001$. Data normalized to housekeeper *36B4.*

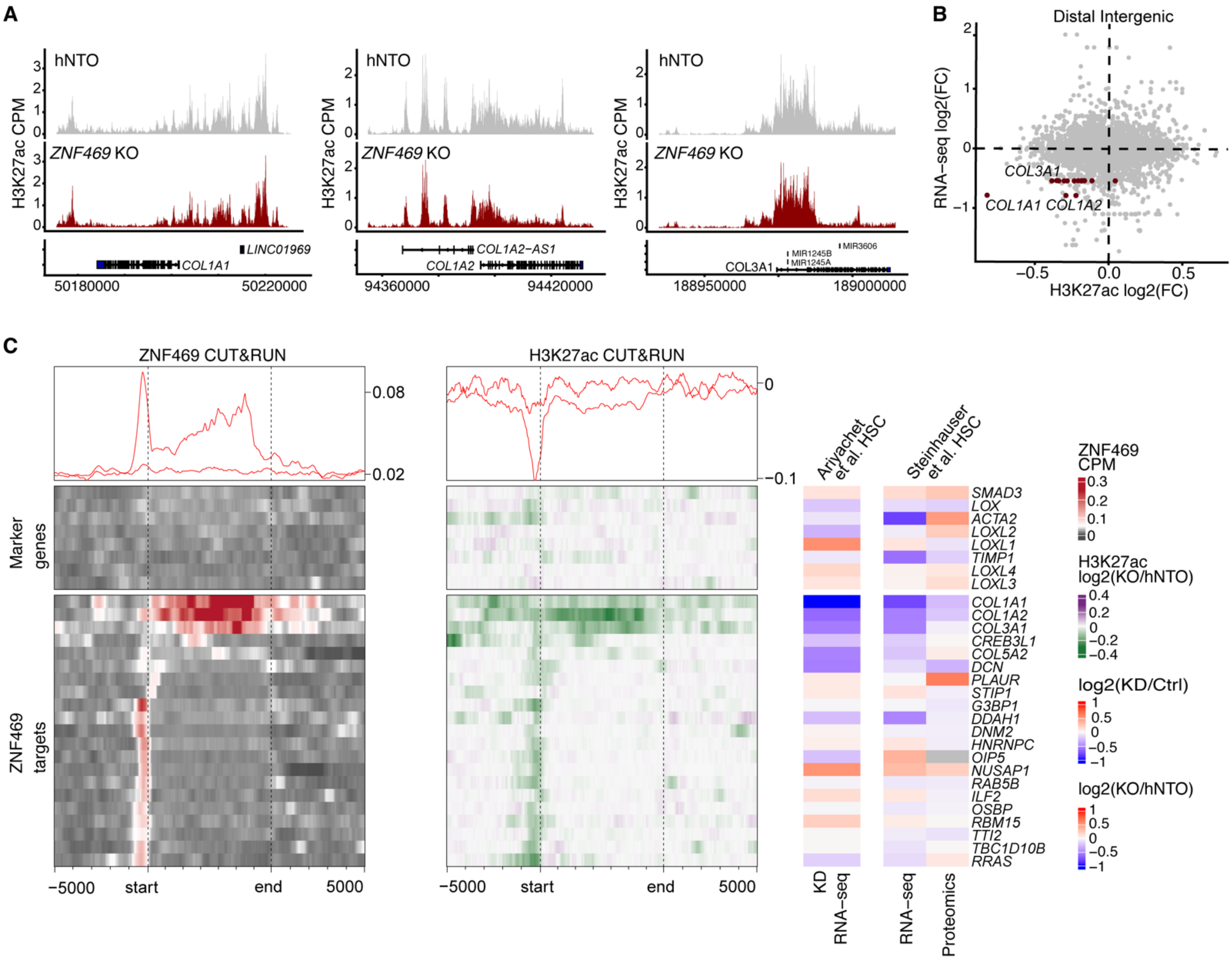

Supplemental Figure 5: ZNF469 knockout alters enhancer signal at collagen and ECM gene loci in HSCs. (**A**) Genome browser showing H3K27ac with or without *ZNF469* KO at *COL1A1*, *COL1A2* and *COL3A1* loci, (**B**) RNAseq and CUT&RUN integration upon *ZNF469* KO highlighting *COL1A1* and *COL1A2* as the top affected genes using distal intergenic regions. **(C)** Heatmaps of CUT&RUN (ZNF469 and H3K27ac) integrated with RNA-seq (both published and using data generated in Figure 3) and unbiased proteomics with a focus on hepatic stellate marker expression.

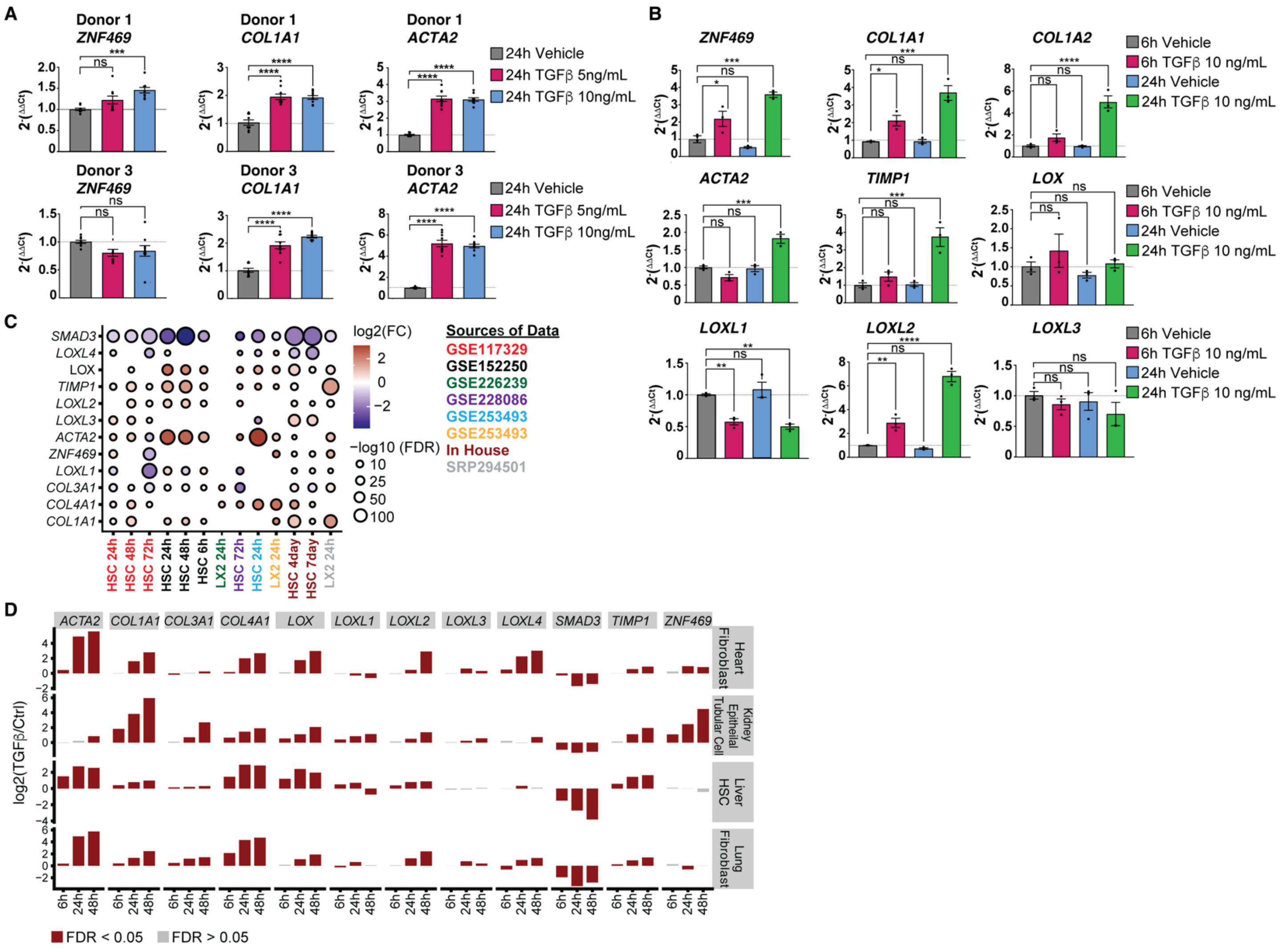

Supplemental Figure 6. Expression of *ZNF469* and other markers of HSC activation in response to TGF-β. Bar plots of gene expression from (A) 2 Donors from HSCs, **(B)** LX-2 cells, **(C)** public and in-house data in liver, **(D)** fibroblasts in heart, kidney, liver and lung from public study GSE152250. For (A,B) p-values determined by one-way ANOVA and Dunnett's multiple comparison test. *p<0.05; **p<0.01,***p<0.001, ****p<0.0001. For (A,B) the data are normalized to the housekeeper gene *RPL32.*

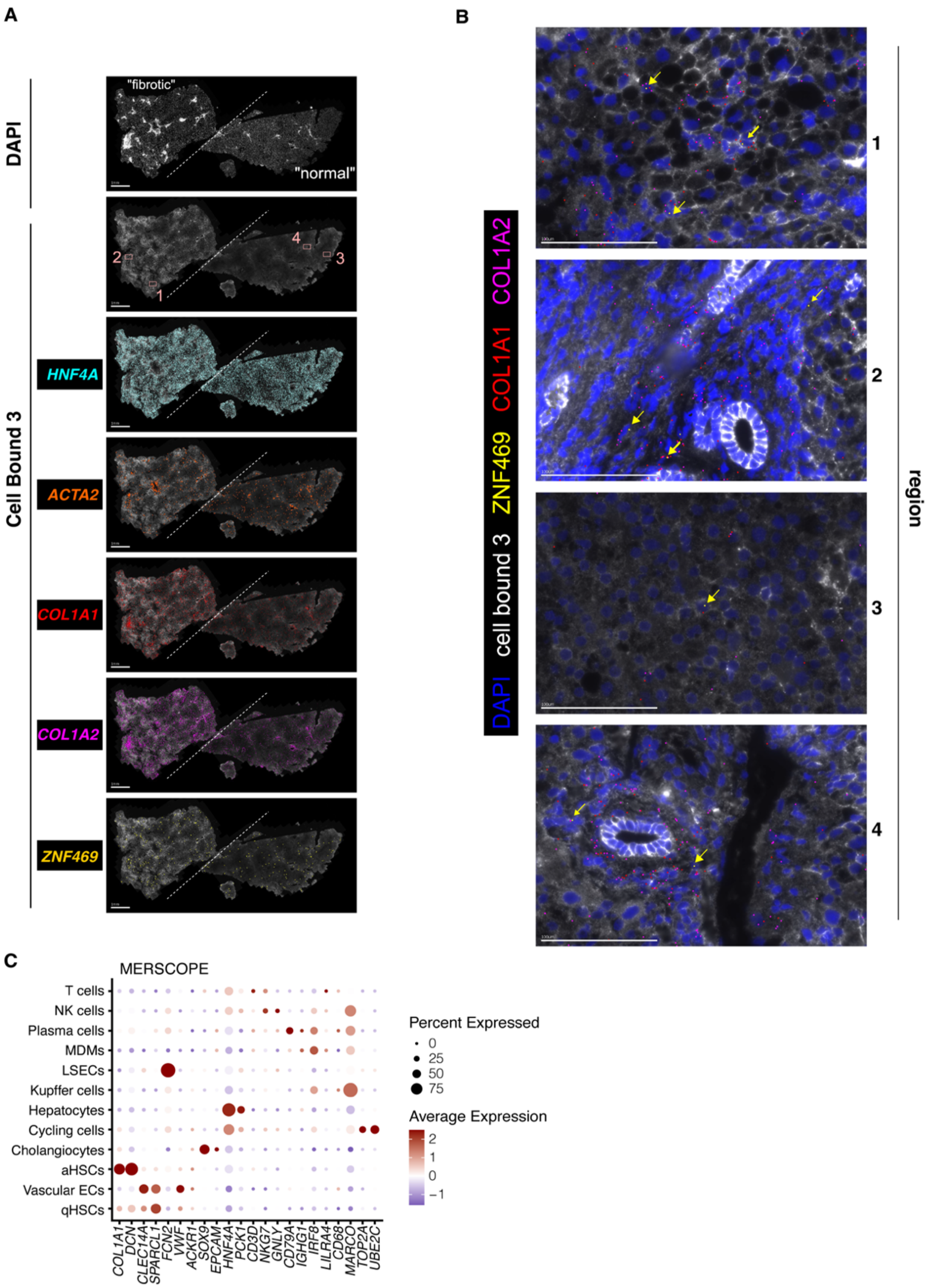

Supplemental Figure 7. ***ZNF469*** **mRNA colocalizes with stellate markers.** Representative MERSCOPE images from liver sections with "fibrotic" and "normal" phenotypes. Dashed line demarcates sections from two distinct donors on the slide. Cell bound 3 stains cell boundaries. Dots represent detected mRNA transcripts color coded for the corresponding gene. (**A**) Low magnification view of tissue sections with selected transcripts. *HNF4A* serves as a hepatocyte marker and technical control. Numbered inlets mark the regions shown in panel B. (**B**) Higher magnification of regions from panel A. DAPI (blue) and cell bound 3 (white) are both shown. Yellow arrows mark *ZNF469* transcripts detected in the given field. (**C**) Marker dot plot per cell type.

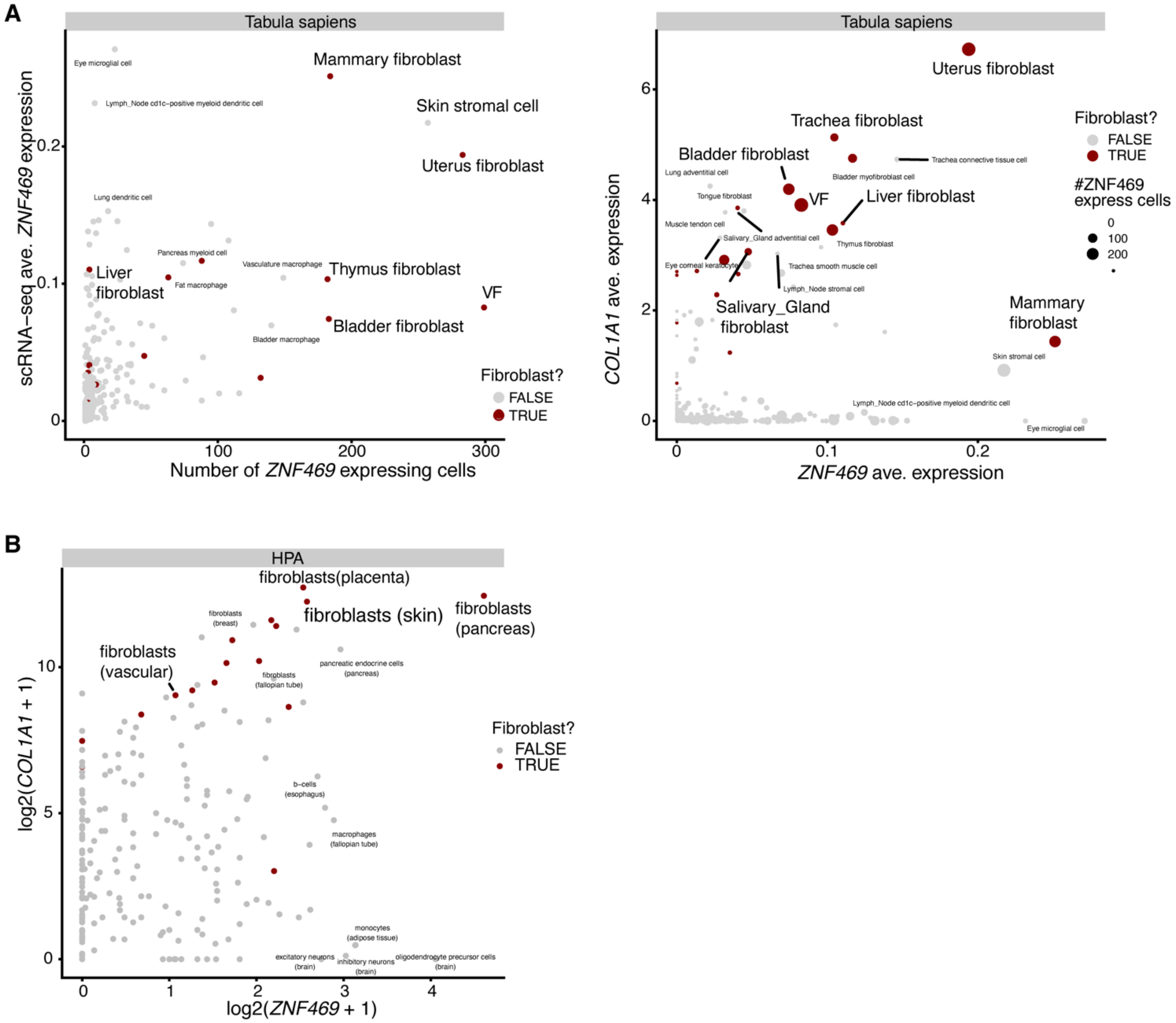

Supplemental Figure 8. ***ZNF469*** **expression is enriched in fibroblasts across multiple tissues.** Public datasets from Tabula Sapiens **(A)** and protein expression from Human Protein Atlas **(B)** were mined for mRNA expression.

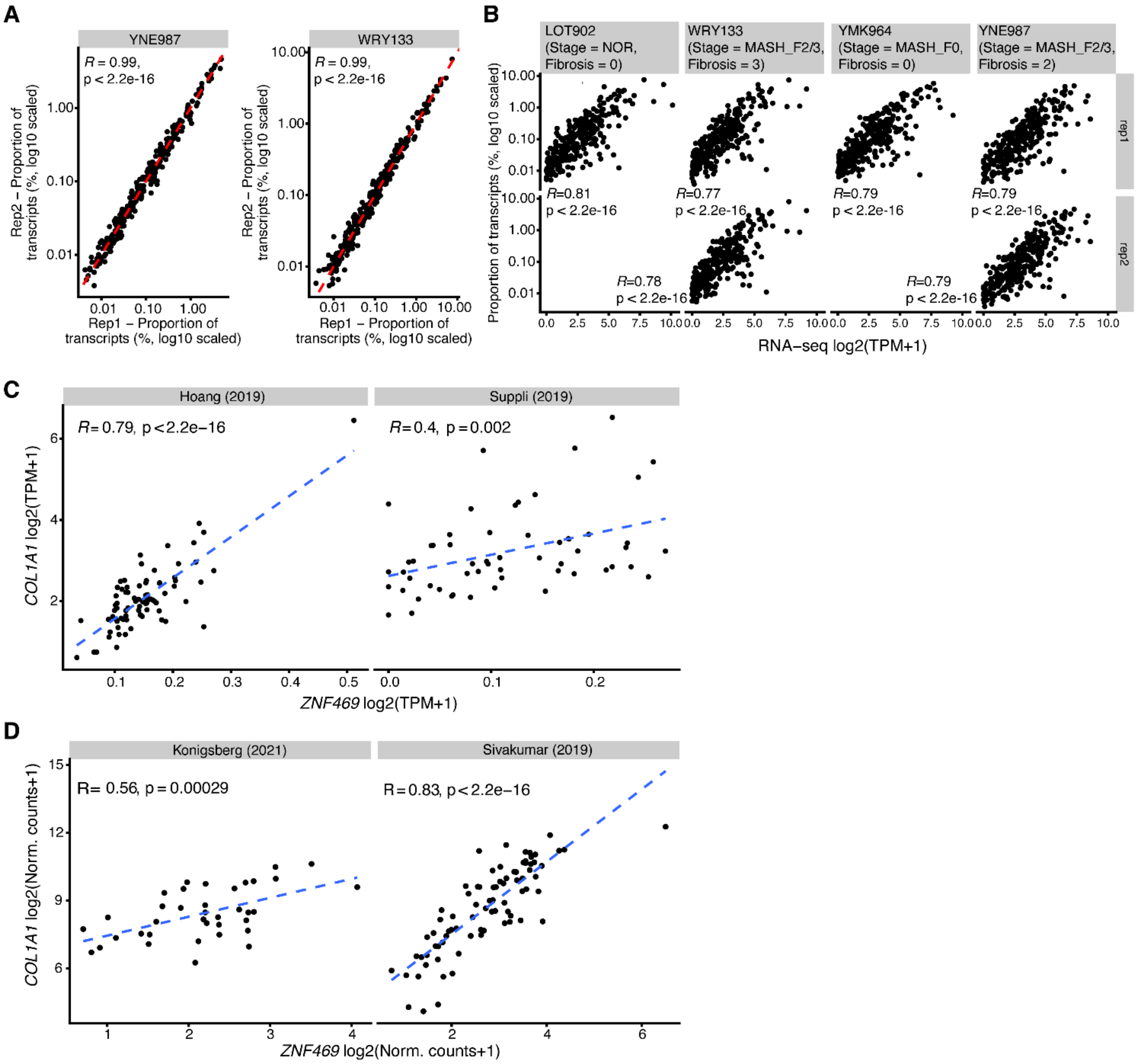

Supplemental Figure 9: ZNF469 expression correlates with collagen production in HSCs in human MASLD. (**A**) MERSCOPE correlation between replicates (**B**) MERSCOPE correlation compared to RNAseq (**C**) Scatter plot showing correlation (Pearson) of *ZNF469* and *COL1A1* expression in public MASH cohorts. (**D**) Scatter plot showing correlation (Pearson) of *ZNF469* and *COL1A1* expression in public interstitial pulmonary fibrosis cohorts.

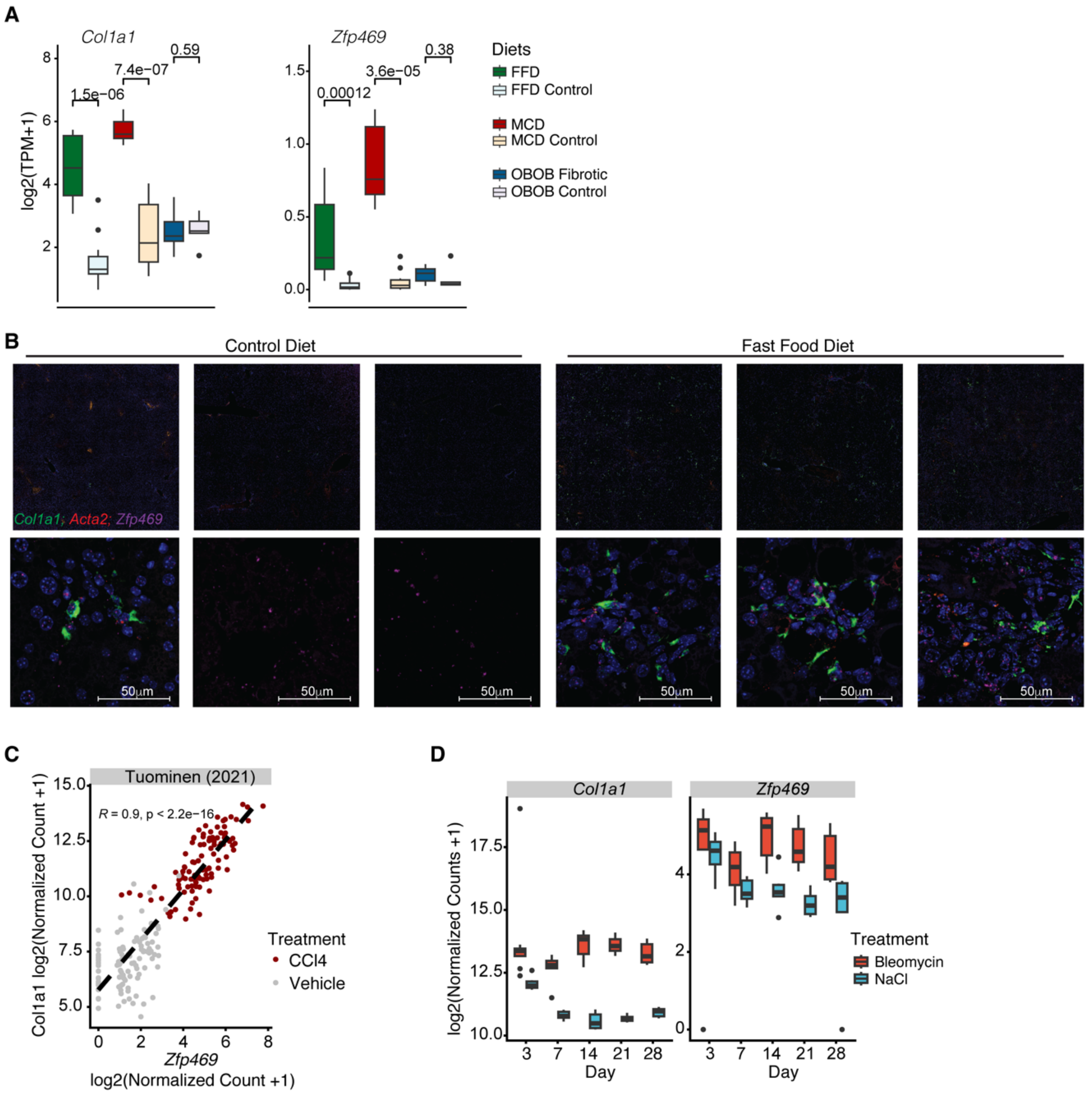

Supplemental Figure 10: *Zfp46*9 expression correlates with collagen in HSCs in mouse models of liver and lung fibrosis. (**A**) Box plots of relative differences in *Zfp469* mRNA in RNA-seq datasets across three different mouse models of liver fibrosis (FFD/GAN diet, methionine-choline deficient diet (MCD) or ethionine-treated ob/ob = fibrotic ob/ob). For FFD and MCD, both male and female mice were used, n=6 per diet condition per sex and data from both sexes are combined to generate the graph. For the ob/ob experiment, male mice were used, n=6, (**B**) RNAscope of *Zfp469* and *Col1a1* co-expression in control diet and GAN diet mouse livers. (**C**) *Zfp46*9 and *Col1a1* expression analyzed from publicly available CCL_4 mouse model (liver), (**D**) *Zfp469* and *Col1a1* expression analyzed from publicly available bleomycin mouse model (lung).
